# Presence of acyl-homoserine lactones in 57 members of the *Vibrionaceae* family

**DOI:** 10.1111/jam.12264

**Published:** 2013-06-28

**Authors:** AA Purohit, J A Johansen, H Hansen, H-KS Leiros, A Kashulin, C Karlsen, A Smalås, P Haugen, NP Willassen

**Affiliations:** 1 The Norwegian Structural Biology Centre, University of TromsøTromsø, Norway; 2 Department of Chemistry, University of TromsøTromsø, Norway

**Keywords:** acyl-homoserine lactones, AHLs, HPLC-MS/MS, quorum sensing, *Vibrionaceae*

## Abstract

**Aims** The aim of this study was to use a sensitive method to screen and quantify 57 *Vibrionaceae* strains for the production of acyl-homoserine lactones (AHLs) and map the resulting AHL profiles onto a host phylogeny.

**Methods and Results** We used a high-performance liquid chromatography–tandem mass spectrometry (HPLC-MS/MS) protocol to measure AHLs in spent media after bacterial growth. First, the presence/absence of AHLs (qualitative analysis) was measured to choose internal standard for subsequent quantitative AHL measurements. We screened 57 strains from three genera (*Aliivibrio*,*Photobacterium* and *Vibrio*) of the same family (i.e. *Vibrionaceae*). Our results show that about half of the isolates produced multiple AHLs, typically at 25–5000 nmol l^−1^.

**Conclusions** This work shows that production of AHL quorum sensing signals is found widespread among *Vibrionaceae* bacteria and that closely related strains typically produce similar AHL profiles.

**Significance and Impact of the Study** The AHL detection protocol presented in this study can be applied to a broad range of bacterial samples and may contribute to a wider mapping of AHL production in bacteria, for example, in clinically relevant strains.

## Introduction

Bacterial cells produce and excrete small molecules into their surroundings to control a number of cellular processes (Ng and Bassler 2009), such as bioluminescence (Meighen 1994), swarming (Eberl *et al*. 1996), biofilm formation, plasmid conjugal transfer (Fuqua *et al*. 1994), production of exo-enzyme virulence determinants in pathogens (Jones *et al*. 1993), antibiotic biosynthesis (Lowery *et al*. 2009), iron chelation (Kaufmann *et al*. 2005) and fouling (Cuadrado-Silva *et al*. 2013). There are currently at least two alternative hypotheses for this behaviour (West *et al*. 2012): (i) quorum sensing (QS), which assumes that the produced molecules are used as signals for cell–cell communication (i.e. a social trait), and (ii) diffusion sensing (DS), which assumes that the produced molecules are used to monitor the rate of diffusion (i.e. the relative speed of molecules that move away from the cell).

Among the different types of such molecules that are produced in bacteria (i.e. autoinducers) the *N*-acyl-homoserine lactones (AHLs) are well studied (Savka *et al*. 2011). AHLs were first studied in *Aliivibrio fischeri* (Eberhard *et al*. 1981), and the bacterium remains a model system for current QS studies (Romero-Campero and Perez-Jimenez 2008). In *A. fischeri*, AinS (a homologue of LuxM in *Vibrio harveyi*) is responsible for the production of C8-homoserine lactone (HSL), whereas LuxI is responsible for the production of 3-oxo-C6-HSL and C6-HSL (Kuo *et al*. 1994). AHL concentrations in bacterial surroundings vary. The squid symbiont *A. fischeri* produces 100 nmol l^−1^ within the squid (Boettcher and Ruby 1995) compared to 0·01–7400 nmol l^−1^ when grown *in vitro* (Stabb *et al*. 2008). Detected AHL levels in cultures of *Pseudomonas aeruginosa* grown *in vitro* are 1–10 μmol l^−1^, whereas 600 μmol l^−1^ is detected in biofilms (Teplitski *et al*. 2011). Lower concentrations of AHL have been detected from mucopurulent respiratory secretions (0·5 pmol l^−1^–66·5 nmol l^−1^) and infected lung tissue (66 fmol g^−1^–146 pmol g^−1^) (Favre-Bonté *et al*. 2002; Chambers *et al*. 2005).

Several bioassays have been used to detect and quantify AHLs. In these assays, the presence of AHL compounds induces phenotypic changes (e.g. bioluminescence or β-galactosidase activity). Bioassays based on *Agrobacterium tumefaciens* and the β-galactosidase reporter system (Cha *et al*. 1998) are triggered by 3-oxo-HSL-induction of the β-galactocidase, and it was recently shown that *A. tumefaciens* was able to detect AHLs as low as 2–14 pmol l^−1^ (Baldrich *et al*. 2011). A second assay based on *Chromobacterium violaceum* produces the purple compound violacein in the presence of short AHLs with no substitutions on the C3 carbon on the acyl chain (McClean *et al*. 1997). Bacterial extracts separated by thin-layer chromatography (TLC) before subjection to a biosensor assay may further discriminate between different AHL types (Ravn *et al*. 2001).

Mass spectrometry (MS) in combination with either gas chromatography (GC) or liquid chromatography (LC) can both be used for detecting and quantifying AHLs. Although GC-MS has the advantage of being a rapid, simple and selective method, it is also associated with unwanted degradation of AHLs during the analysis, which results in low sensitivity (Osorno *et al*. 2012). LC-MS is particularly useful in identifying/quantifying particular molecules in a mixture of different chemicals (Wang *et al*. 2011). The technique has undergone significant development with respect to quantification levels with the utilization/refinement of, for example, nano-LC (Frommberger *et al*. 2004; Fekete *et al*. 2007) and Fourier transform ion cyclotron resonance MS (Cataldi *et al*. 2009, 2013). Although the required volume of samples has declined, it still has remained relatively high. The first AHL study used 6-l sample volumes for extraction of AHLs (Eberhard *et al*. 1981); later it was reduced to 500 ml (Shaw *et al*. 1997), 20 ml (Yates *et al*. 2002) and 500 μl (Frommberger *et al*. 2004). In this work, we have used 75-μl sample volume, with a sample processing rate on the LC-MS machine at >200 samples per day. We also wanted to make the method affordable, so isotopically labelled internal standards were excluded.

The *Vibrionaceae* family consists of Gram-negative marine bacteria, many of which are responsible for diseases in humans (Valiente *et al*. 2009) and marine animals (Milton 2006), and AHLs of QS systems likely play important roles in pathogenesis (Hentzer and Givskov 2003; Rasch *et al*. 2004; Nhan *et al*. 2010). In this study, we used a high-performance liquid chromatography–tandem mass spectrometry (HPLC-MS/MS) protocol in selective reaction monitoring (SRM) mode for determining the presence/absence (qualitative analysis) and concentrations of fifteen different AHLs and applied this method to spent media. We tested our method on *A. fischeri* ES114 to compare with previous data (Lupp and Ruby 2004; Stabb *et al*. 2008). We screened 7 *Vibrio*, 5 *Photobacterium* and 45 *Aliivibrio* isolates and included among others *Vibrio anguillarum*,*Vibrio splendidus, Aliivibrio salmonicida, A. fischeri*,*Aliivibrio wodanis*,*Aliivibrio logei* and *Photobacterium phosphoreum*. AHL measurements were finally mapped onto a host phylogeny to evaluate whether closely related species and strains produce similar AHL profiles.

## Materials and methods

### Chemicals and AHL standard mixture

The AHL standards *N*-3-oxo-butyryl-l-homoserine lactone (3-oxo-C4-HSL), *N*-3-hydroxy-butyryl-l-homoserine lactone (3-OH-C4-HSL), *N*-3-hydroxy-hexanoyl-l-homoserine lactone (3-OH-C6-HSL), *N*-3-hydroxy-octanoyl-l-homoserine lactone (3-OH-C8-HSL) and *N*-3-hydroxy-decanoyl-l-homoserine lactone (3-OH-C10-HSL) were purchased from University of Nottingham, UK, whereas *N*-butyryl-dl-homoserine lactone (C4-HSL), *N*-hexanoyl-l-homoserine lactone (C6-HSL), *N*-3-oxo-hexanoyl-l-homoserine lactone (3-oxo-C6-HSL), *N*-octanoyl-l-homoserine lactone (C8-HSL), *N*-3-oxo-octanoyl-l-homoserine lactone (3-oxo-C8-HSL), *N*-decanoyl-dl-homoserine lactone (C10-HSL), *N*-3-oxo-decanoyl-l-homoserine lactone (3-oxo-C10-HSL), *N*-dodecanoyl-dl-homoserine lactone (C12-HSL), *N*-3-oxo-dodecanoyl-l-homoserine lactone (3-oxo-C12-HSL), *N*-3-hydroxy-dodecanoyl-dl-homoserine lactone (3-OH-C12-HSL) and ethyl acetate were purchased from Sigma-Aldrich. HPLC-grade acetonitrile (LiChrosolv) and formic acid were purchased from Merck. The AHL standard mixture contained approximately 600–1200 nmol l^−1^ of each AHL in 0·1% formic acid.

### Bacterial growth

*Aliivibrio fischeri* ES114 was revived from a cryopreserved glycerol stock in 2 ml lysogeny broth (LB; 1 l contains 10 g bacto-tryptone, 5 g yeast extract and 10 g NaCl) supplied with 2·5% w/v sodium chloride (LB25; 1 l contains 10 g bacto-tryptone, 5 g yeast extract and 25 g NaCl) at 12°C, 200 rpm (Infors – Incubator Shaker Multitron II) for 24–48 h. Experimental cultures were further expanded at 12, 22 and 30°C, initiated by inoculating 50 ml of LB25 or sea water tryptone (SWT; 1 l contains 5 g bacto-tryptone, 3 g yeast extract, 3 ml glycerol, 700 ml filtered sea water and volume made to 1000 ml with filtered water) in 250-ml baffled flasks. When using SWT during the initial study, samples were collected at several optical densities, whereas when using LB25 during the screening, the culture was harvested after 50 h of growth and treated as described below.

Strains listed in Table 1 were grown in duplicates at 12°C in LB25 (as described above). The new strains reported in this study are from an in-house bacterial collection. Starting cultures were inoculated at OD_600_ 0·001 and harvested after 50 h of growth. Samples were centrifuged at 1400 ***g*** in a Beckman Coulter Allegra X-15R tabletop centrifuge with SX4750A rotor for 20 min at 4°C. Four 75-μl aliquots (technical replicates) were acidified with 4 μl 1 mol l^−1^ hydrochloric acid to a final concentration of 51 mmol l^−1^ and stored at −20°C. One replicate was used for qualitative analysis, and the remaining three were used for quantitative analysis (described below).

**Table 1:** Bacterial species tested for AHL production

Genera; species	Strain	Origin	GenBank	Reference
*Aliivibrio*				
*A. fischeri*	ES114	*Euprymna scolopes*	FJ464360	Gutierrez *et al*. (2009)
*A. logei*	03/SES-5	*Gadus morhua*	JQ361740	This study
*A. logei*	03/SES-1	*Gadus morhua*	JQ361739	This study
*A. logei*	ATCC 29985	Gut of *Mytilus edulis* (arctic mussel)	EU221273	Ast *et al*. (2009)
*A. logei*	90/1667	*Salmo salar*	JQ361738	This study
*A. salmonicida*	LFl1238	*Salmo salar*	FM178379	Hjerde *et al*. (2008)
*A. salmonicida*	NCIMB 2262 (T)	Farmed *Salmo salar*, Norway	EU257756	Brevik *et al*. (2007)
*Aliivibrio* sp.	R5-43	*Gersemia rubiformis (soft coral)*	JQ340770	This study
*Aliivibrio* sp.	R5-42	*Gersemia rubiformis (soft coral)*	JQ361697	This study
*Aliivibrio* sp.	R8-70	*Eurythenes gryllus (Amphipoda)*	JQ361698	This study
*Aliivibrio* sp.	R8-66	*Eurythenes gryllus (Amphipoda)*	JQ361699	This study
*Aliivibrio* sp.	R8-65	*Eurythenes gryllus (Amphipoda)*	JQ361700	This study
*Aliivibrio* sp.	R8-61	*Eurythenes gryllus (Amphipoda*	JQ361701	This study
*Aliivibrio* sp.	R8-69	*Eurythenes gryllus (Amphipoda)*	JQ361702	This study
*Aliivibrio* sp.	R8-68	*Eurythenes gryllus (Amphipoda)*	JQ361703	This study
*Aliivibrio* sp.	B8-7	*Eurythenes gryllus (Amphipoda)*	JQ361704	This study
*Aliivibrio* sp.	R8-63	*Eurythenes gryllus (Amphipoda)*	JQ361705	This study
*Aliivibrio* sp.	R8-67	*Eurythenes gryllus (Amphipoda)*	JQ361706	This study
*Aliivibrio* sp.	R8-64	*Eurythenes gryllus (Amphipoda)*	JQ361707	This study
*Aliivibrio* sp.	MR17-66	*Styela rustica (Ascidiacea)*	JQ361710	This study
*Aliivibrio* sp.	MR17-80	*Porifera indet*	JQ361711	This study
*Aliivibrio* sp.	MR17-77	*Porifera indet*	JQ361712	This study
*Aliivibrio* sp.	MR17-69	*Styela rustica (Ascidiacea)*	JQ361713	This study
*Aliivibrio* sp.	MR17-34	*Laminaria sp*.	JQ361714	This study
*Aliivibrio* sp.	MR17-70	*Styela rustica (Ascidiacea)*	JQ361715	This study
*Aliivibrio* sp.	B9-15	*Dendrodoa aggregata (Ascidiacea)*	JQ361716	This study
*A. wodanis*	89/5532	*Salmo salar*	JQ361718	This study
*A. wodanis*	90/325	*Salmo salar*	JQ361719	This study
*A. wodanis*	02/569	*Salmo salar*	JQ361720	This study
*A. wodanis*	02/382	*Salmo salar*	JQ361721	This study
*A. wodanis*	01/401	*Salmo salar*	JQ361722	This study
*A. wodanis*	88/441 (T)	*Salmo salar*	JQ361723	This study
*A. wodanis*	06/194-A	*Salmo salar*	JQ361724	This study
*A. wodanis*	06/194-B	*Salmo salar*	JQ361725	This study
*A. wodanis*	06/139	*Salmo salar*	JQ361726	This study
*A. wodanis*	06/170	*Salmo salar*	JQ361727	This study
*A. wodanis*	96/688	*Salmo salar*	JQ361728	This study
*A. wodanis*	03/160	*Salmo salar*	JQ361729	This study
*A. wodanis*	06/178	*Oncorhynchus mykiss*	JQ361731	This study
*A. wodanis*	04/17347	*Gadus morhua*	JQ361730	This study
*A. wodanis*	SR6	*Sepiola robusta*	EU185827	Ast *et al*. (2009)
*A. wodanis*	SA12	*Sepiola affinis*	EU185826	Ast *et al*. (2009)
*A. wodanis*	ATCCBAA104 (T)	*Salmo salar*	EU257757	Brevik *et al*. (2007)
*Photobacterium*				
*P. phosphoreum*	SP001	Gut of *Cebidichthys violaceus* (Family Stichaeidae)	JX312085	This study
*P. phosphoreum*	SP002	Gut of *Lycodes eudipleurostictus*	JX312086	This study
*P. phosphoreum*	SP004	Gut of *Cottunculus microps*	JX312087	This study
*P. phosphoreum*	SP005	Gut of *Onogadus argentatus*	JX312088	This study
*Photobacterium* sp.	SP0044	Marine sediment	JX312089	This study
*Vibrio*				
*V. anguillarum*	NB10	*Gadus morhua*	DQ068933	Rehnstam *et al*. (1989), Eiler and Bertilsson (2006)
*Vibrio* sp.	B9-25	*Halichondria sp. (demosponge)*	JQ361717	This study
*V. splendidus*	03/122	*Hippoglossus hippoglossus*	JQ361735	This study
*V. splendidus*	00/860	*Labrus mixtus*	JQ361736	This study
*V. splendidus*	02/066	*Gadus morhua*	JQ361733	This study
*V. splendidus*	04/276	*Gadus morhua*	JQ361734	This study
*V. splendidus*	02/14916	*Gadus morhua*	JQ361732	This study
*V. splendidus*	LMG 19031 (T)	*Gadus morhua*	EF094892	Gomez-Gil *et al*. (2006)
*V. tapetis*	99/196	*Oncorhynchus mykiss*	JQ361737	This study

T, type strain; ATCC, American Type Culture Collection; LMG, Belgium Co-ordinated Collection of Micro-organisms, Laboratorium voor Microbiologie, Universiteit Gent, Belgium; NCIMB, National Culture of Industrial, Marine and Food Bacteria, Aberdeen, Scotland.

*Escherichia coli* ArcticExpress (DE3) was grown overnight at 30°C in LB medium.

### Sample preparation and HPLC-MS/MS analysis

The concentration of AHLs in spent media was measured as follows: AHLs were extracted from spent media and compared to commercial AHL standards with known concentrations which were extracted from fresh media using the same extraction protocol, as described below. Hence, it was the relative relationships between measured AHLs from spent media and the corresponding values from commercial AHLs that were used to calculate the final AHL concentrations in samples.

Four acidified 75-μl technical replicates, which were collected at each time point or OD, were each subjected to three consecutive extractions with three volumes (225 μl) of ethyl acetate. The ethyl acetate phase from the three extractions was pooled (675 μl) and dried using a rotary vacuum centrifuge (savant instruments Inc. model PH40-11), and samples were finally stored at −20°C for subsequent HPLC-MS/MS analysis. One technical replicate was redissolved in 150 μl of 20% acetonitrile with 0·1% formic acid for qualitative HPLC-MS/MS analysis. Similarly, three technical replicates were redissolved in 20% acetonitrile with 0·1% formic acid, which contained a fixed amount of an internal AHL standard [100 ng/ml (i.e. 330–400 nmol l^−1^ depending on the molecular weight of the AHL]. HPLC was performed by separating 20 μl of the 150 μl from each sample on a Hypersil GOLD C18 reverse-phase column (50 × 2·1 mm, 1·9 μm particle size; Thermo Fisher Scientific, Waltham, MA, USA) equilibrated with 5% acetonitrile, using a Accela autosampler and pump (Thermo Fisher Scientific). Mobile phase was 0·1% formic acid in water and 0·1% formic acid in HPLC-grade acetonitrile, and these were used to create the following gradient elution profile: 5% acetonitrile for 18 s, a linear increase in acetonitrile to 90% over 162 s and then isocratic 90% acetonitrile for 60 s. Then in next 60 s, the column was equilibrated with 5% acetonitrile. The column was then re-equilibrated for 60 s with 5% acetonitrile before the next sample injection. The flow rate was 500 μl min^−1^ for all steps, and the column was kept at 30°C for all separations.

**Figure 1:**
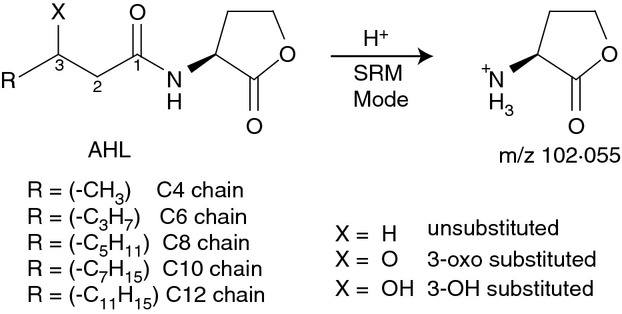
Basic chemical structure of AHLs and their fragmentation products produced during tandem MS in SRM mode. Chain lengths of AHLs screened in this study were from C4 to C12 with an increment of two carbons. Functional groups of the third carbon were 3-oxo, 3-OH or unsubstituted.

The HPLC-separated compounds were introduced into the Ion Max electrospray ionization probe of the LTQ Orbitrap XL machine (Thermo Fisher Scientific). The probe was equipped with a 32-gauge stainless steel needle. Nitrogen was used for all type of gasses in the ion source. Electrospray settings were as follows: sheath gas flow rate 70, auxiliary gas flow rate 10, sweep gas flow rate 10, spray voltage +4·50 kV, capillary temperature 330°C, capillary voltage 40 V and tube lens 90 V. The protonated parent ions from the electron spray were next selected and fragmentized with CID using LTQ (Linear Ion Trap Quadrupole) tandem MS in SRM mode under positive ion conditions. When subjected to tandem MS, all AHLs are broken down to the same fragment with the theoretical value m/z 102·055. This fragment contains only the head (lactone) of the AHL (see Fig. 1). CID parameters during tandem MS were as follows: isolation width = 1·7, normalized collision energy = 30, act Q = 0·250 and act time = 30 ms for all components. Maximum injection time was 25 ms, and automatic gain control (AGC) target setting is 1·00E+04 ions.

SRM mode involves selecting a parent ion and breaking it with a constant energy to give fragment ions. One such parent–fragment pair is called an SRM transition. To minimize the number of simultaneous SRM transitions and maximize the number of data points over each peak, the data collection window was divided into 6 segments (for details see Table S1). Data were collected from m/z 101–103. Ion chromatograms were extracted from the raw data files from m/z 101·8–102·6 for each compound (see Fig. S1 for typical ion chromatograms). The peaks were automatically integrated with optimized integration settings for each compound using Thermo Excalibur 2·07. For the entire study, one-point calibration was used (instead of using standard curves), as previously described (Peters and Maurer 2007). One-point calibration was used for each purchased standard AHL. Calibration was performed in triplicates for each sample run, in same growth media as in samples.

In quantitative analyses, an internal standard was added to all biological samples and AHL standard mixture in equal amount to correct for deviations in injection volume of the autosampler and to correct for changes in the instrument sensitivity. 3-oxo-C12-HSL, 3-OH-C12-HSL, C10-HSL and C12-HSL were chosen as internal standards depending on the test sample. The AHL standard mixture was made fresh for each run.

The limit of detection (LOD) and limit of quantification (LOQ) were determined (LOQs listed in Table 2) from the mix of synthetic AHLs serially diluted into growth medium to a minimum concentration of 0·2 nmol l^−1^, extracted and subjected to HPLC-MS/MS analysis. LOD was defined as the lowest AHL concentration where the signal-to-noise ratio was ≥ 3 : 1 (MacDougall *et al*. 1980), and LOQ was theoretically set to LOD multiplied by 3·33 (Thomsen *et al*. 2003).

**Table 2:** Limit of AHL quantification for HPLC-MS/MS (SRM) method

AHLs	LOQ (nmol l^−1^) LB25	LOQ (nmol l^−1^) SWT	LOQ (nmol l^−1^) LB	LOQ (nmol l^−1^) H_2_O
C4-HSL	12·92	12·92	12·92	12·92
3-oxo-C4-HSL	20·06	11·27	20·06	6·33
3-OH-C4-HSL	19·85	11·15	19·85	19·85
3-OH-C6-HSL	5·99	3·20	5·99	3·37
3-oxo-C6-HSL	10·35	3·27	5·82	5·82
C6-HSL	3·06	1·63	3·06	3·06
3-oxo-C8-HSL	9·37	5·01	9·37	5·27
3-OH-C8-HSL	2·98	2·68	2·98	1·67
C8-HSL	3·23	1·81	3·23	1·81
3-oxo-C10-HSL	2·54	2·54	2·54	2·54
3-OH-C10-HSL	1·57	0·88	0·88	0·88
C10-HSL	1·58	2·82	1·58	0·89
3-OH-C12-HSL	1·35	1·35	2·40	0·76
3-oxo-C12-HSL	1·29	4·08	1·29	4·08
C12-HSL	4·80	1·51	1·51	1·51

AHL, Acyl-homoserine lactones; LOQ, limit of quantification.

Calculated as 3·33 times of LOD values (signal-to-noise ratio better than 3:1), practically its values with signal-to-noise ratio are greater than 10:1.

### Phylogenetic analysis

The host phylogeny was based on 16S rDNA sequences from forty-five *Vibrionaceae* strains, and *Photorhabdus luminescens* and *Grimontia hollisae* were used as the out-group. Available 16S rDNA sequences were obtained from GenBank, and for the remaining strains, 16S rDNA was PCR-amplified and sequenced. Accession numbers are provided in Table 1. The partial 16S rDNA sequences were aligned automatically by ClustalW (Larkin *et al*. 2007) and manually edited in BioEdit v.7·0·9 (Hall 1999). Sequence positions that could be reliably aligned were used, and the final alignment consisted of 852 nucleotide positions and 45 sequences (Dataset S1). The phylogenetic tree was constructed in MEGA v.5.03 (Tamura *et al*. 2011) using the neighbour-joining (NJ) method and the maximum composite likelihood substitution model. Stability of nodes was tested with a NJ bootstrap analysis (maximum composite likelihood model and 10 000 pseudo-replicates).

## Results

### AHL detection method

HPLC followed by tandem MS in SRM mode was used to measure concentrations of AHLs. As a standard protocol, HPLC was run with 5 min from injection to elution and 1-min column equilibration before next injection. To minimize the number of simultaneous SRM transitions and maximize the number of data points over each peak, the data collection window was divided into 6 segments (for details see Table S1).

Before running biological samples, we first determined the LOD and LOQ. A mix of synthetic AHLs was serially diluted in water or growth medium (LB, LB25 or SWT) to a minimum concentration of 0·2 nmol l^−1^, extracted and subjected to our HPLC-MS/MS protocol (see Material and methods). Three replicates were performed for each test concentration. LOD was defined as the lowest AHL concentration where the signal-to-noise ratio was ≥ 3 : 1 (MacDougall *et al*. 1980), and LOQ was theoretically set to LOD multiplied by 3·33 (Thomsen *et al*. 2003). Table 2 shows the LOQ values. For C4-chained AHLs and longer-chained AHLs, LOQ ranged between 6·33–20·06 and 0·76–10·35 nmol l^−1^, respectively. In other words, to reliably quantify, for example, C4-chained AHLs, the AHL concentration in a sample must be ≥ 6·33–20·06 nmol l^−1^ depending on which medium is used.

Next, the linearity of AHL measurements was tested over a broad range of concentrations (15–3000 nmol l^−1^), in water or same media as described above. Measurements were plotted, and resulting R^2^ values between 0·9781 and 0·9996 (for LB, LB25, SWT media and H_2_O – only LB25 data are shown in Fig. S2) demonstrated linear relationships between expected AHL concentrations and experimental measurements. Accuracy (i.e. deviation from the true value) was determined by adding 14 synthetic AHLs (C10-HSL was arbitrarily chosen as the internal standard) at three different concentration ranges (15–30, 900–1800 and 1300–2700 nmol l^−1^) to a supernatant of *Escherichia coli* ArcticExpress (DE3) overnight culture (Table S2). Samples were subjected to HPLC-MS/MS. This strain was chosen because it does not carry AHL-producing enzymes (Van Houdt *et al*. 2006). The majority of values were within 10% of the expected concentrations, with three exceptions in lowest concentration range (15–30 nmol l^−1^), that is, C4-HSL (expected/detected=31 : 42 nmol l^−1^), 3-OH-C6-HSL (25 :22 nmol l^−1^) and 3-OH-C8-HSL (23 : 16 nmol l^−1^). Similarly, there were two deviations above 10% for medium and higher concentration ranges, that is, 3-OH-C4-HSL (1568 : 1410 and 2352 : 2028 nmol l^−1^) and 3-oxo-C6-HSL (2180 : 1860 nmol l^−1^). We suspect that major contributors to these variations are measurement errors (especially at low AHL concentrations) and degradation of the analytical standards. Measurement errors would include variations between technical replicates (random error) and lowered accuracy because all AHLs are compared to one single internal standard (systematic error). The most important systematic error in electrospray MS is the phenomenon known as ion suppression, where matrix components eluting at the same time as the analyte suppress the signal of the analyte. Random errors could be minimized by increasing the number of replicates. Ion suppression could be minimized by several approaches like better sample preparation, running standards in the exact same matrix as the analyte (performed as close as possible), altered chromatographic profile, diluting the sample, changing the mobile phase's organic solvent, spiking the sample with standards (time-consuming) and so on. The best method is likely to use one stable isotopically labelled internal standard (SIL) for each AHL. The SIL should preferably have the exact same retention time as the analyte to be exposed to the same random spray variations and ion suppression effects. Deuterium labelling might give slightly different retention times, therefore ^13^C-, ^15^N- or ^17^O-labelled standards are preferred. These are, however, usually unavailable or very expensive.

Finally, the precision (as relative standard deviation) of the method was calculated using the same data as described above, to test the reproducibility of the detected value (Table S2). Precision ranged between 0·1 and 13·0% with four values above 10%, that is, 3-oxo-C4-HSL (13%), 3-oxo-C6-HSL (12%), 3-OH-C8-HSL (11%) and 3-OH-C4-HSL (10%). This shows that results were, in general, reproducible (or precise) between replicate measurements and that AHLs were successfully recovered from bacterial culture supernatants in a reproducible fashion.

### AHLs in *Aliivibrio fischeri* ES114

Next we wanted to test our method using the model bacterium *A. fischeri* ES114 and compare AHL measurements with previous results (Stabb *et al*. 2008). For cultivation of *A. fischeri* ES114, we chose SWT medium and three different temperatures, that is, 12, 22 and 30°C. For each temperature, two cultures (biological replicates) were started at OD_600_ 0·05, and samples were collected at different optical densities. Using 3-oxo-C12-HSL as internal standard [based on qualitative HPLC-MS/MS (SRM) analysis], the concentration of individual AHLs from the resulting growth culture supernatants was measured in triplicates. Table 3 shows the result which suggests that *A. fischeri* ES114 typically contains six AHLs (when grown in SWT medium) above LOQ, with highest concentrations of 3-oxo-C6-HSL and C8-HSL. Interestingly, the 3-oxo-C6-HSL profile was highly dependent on temperature. For example, at 12°C, 3-oxo-C6-HSL was found at 670 ± 120 and 1380 ± 320 nmol l^−1^, at OD_600_ ∼0·5 and ∼1·0, respectively, but was found below LOQ when cells were grown at 30°C (although below LOQ, our measurements suggest approx. 0·15 nmol l^−1^ at OD_600_ ∼1·5, data not shown). In contrast, concentrations of C8-HSL appeared less dependent on temperature. Other AHLs that were detected above LOQ include C4-HSL, C6-HSL, 3-OH-C8-HSL and C10-HSL.

**Table 3:** AHL concentrations in spent media from *Aliivibrio fischeri* ES114

Sample (OD_600_)	Temp	C4-HSL (nmol l^−1^)	3-oxo-C6-HSL (nmol l^−1^)	C6-HSL (nmol l^−1^)	3-OH-C8-HSL (nmol l^−1^)	C8-HSL (nmol l^−1^)	C10-HSL (nmol l^−1^)	Reference
0·49 ± 0·03	12°C	62 ± 2	670 ± 120	22 ± 4	–	80 ± 20	–	This study
1·00 ± 0·02	12°C	79 ± 4	1380 ± 320	75 ± 5	5·9 ± 0·7	810 ± 50	17 ± 1	This study
0·66 ± 0·00	22°C	68 ± 2	20 ± 3	2·80 ± 0·30	–	52 ± 3	–	This study
1·65 ± 0·05	22°C	81 ± 3	100 ± 45	12 ± 1	5·1 ± 0·5	242 ± 18	5·2 ± 0·3	This study
2·5 ± 0·1	22°C	96 ± 7	190 ± 82	34 ± 2	14 ± 2	744 ± 120	15 ± 2	This study
1·16 ± 0·08	30°C	65 ± 8	–	2·7 ± 0·3	–	91 ± 6	2·3 ± 0·3	This study
1·56 ± 0·02	30°C	70 ± 8	–	4·8 ± 0·2	–	160 ± 10	4·1 ± 0·4	This study
0·5	?		0·01			35		Stabb *et al*. (2008)
1·5	?		0·1			1100		Stabb *et al*. (2008)
2·8	?		0·2					Stabb *et al*. (2008)
∼0·5	28°C					∼2		Lupp and Ruby (2004)
∼1·5	28°C					∼20		Lupp and Ruby (2004)
∼3·5	28°C					∼80		Lupp and Ruby (2004)

The tandem MS SRM method was used for quantitative analysis with internal standard (3-oxo-C12-HSL). Values are given as nmol l^−1^ of AHLs calculated against one-point calibration of known standards. Two parallel colonies were grown and harvested at different optical densities.

Tested samples were grown at 12, 22 and 30°C in SWT medium. Cultures were grown from OD_600_ 0·05. –, values below LOQ. ?, unknown growth temperature.

Ruby and co-workers (Stabb *et al*. 2008) reported two AHLs in *A. fischeri* ES114, that is, 3-oxo-C6-HSL and C8-HSL. 3-oxo-C6-HSL was measured at 0·01, 0·1 and 0·2 nmol l^−1^ at OD_595_ 0·5, 1·5 and 2·8, respectively, which is very similar to what we found at 30°C, but several orders of magnitude lower than our measurements at 12 and 22°C. Furthermore, Stabb *et al*., reported C8-HSL at 35 nmol l^−1^ at OD_595_ 0·5, which is in close agreement with our measurement (80 ± 20 and 53 ± 3 at 12 and 22°C, respectively), but at OD_595_ = 1·5, C8-HSl was measured at 1100 nmol l^−1^. Lupp and Ruby (Lupp and Ruby 2004) measured C8-HSL using a bioassay, also using *A. fischeri* ES114. After growth in SWT medium at 28°C, they reported approximately 2, 20 and 80 nmol l^−1^ at OD_600_ ∼0·5, ∼1·5 and ∼3·5. In summary, we conclude that *A. fischeri* ES114 may contain six different AHLs, typically with 3-oxo-C6-HSL and C8-HSL at highest concentrations. Measurements of 3-oxo-C6-HSL and C8-HSL appear to be in general agreement with previous data (Lupp and Ruby 2004; Stabb *et al*. 2008), although the concentration of AHLs can greatly vary depending on growth parameters.

### Screening of 57 *Vibrionaceae* strains for AHL production

**Figure 2:**
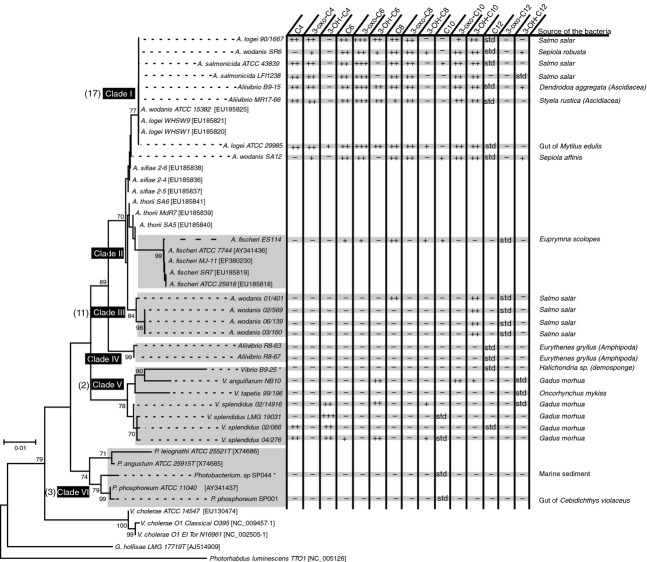
Detected AHLs mapped onto a 16S rDNA ML phylogeny. Bootstrap values above internal nodes were calculated using the NJ method and the maximum composite likelihood model. Clades containing strains that were tested for AHL production are named Clades I–VI. GenBank accession numbers are shown in brackets for species that were added to increase the phylogenetic diversity in the tree. Numbers in parentheses to the left of Clades I–VI denote the number of strains in each clade that were tested, but excluded from the final tree because of redundancy in the data. Asterisks denote strains that grew slowly with a final OD_600_=0·1 when harvested after 50 h of growth. Complete list of results is shown in Table S3. Concentrations of AHLs are grouped into four categories: ‘high’ >5 μmol l^−1^ (+++), ‘medium’ 25–5000 nmol l^−1^ (++), ‘low’<25 nmol l^−1^ (+) and values below LOQ (-).

Following the results from *A. fischeri* ES114, we screened an additional 56 *Vibrionaceae* strains, which were grown in two biological replicates in LB25 at 12°C and harvested after 50 h of growth. LB25 was used for convenience and because this medium could support growth of all tested strains. (The majority of tested strains are of Arctic or sub-Arctic marine origin.) AHLs were measured in three technical replicates per biological replicate using our HPLC-MS/MS protocol, and AHL concentrations were compared to known standards. Variations between biological replicates were generally low (Table S3). For simplicity, the final AHL measurements were organized into three categories, that is, ‘high’ (>5000 nmol l^−1^), ‘medium’ (25–5000 nmol l^−1^) and ‘low’ (<25 nmol l^−1^) and mapped accordingly onto a 16S rDNA host phylogeny (Fig. 2). Data from 24 strains are presented, whereas the remaining 33 were excluded from the figure because of redundancy in the data (listed in Table S4). Twenty-one nontested strains were included in the tree as reference strains to increase the phylogenetic diversity in the data set.

Figure 2 shows that (i) tested strains (except *Aliivibrio wodanis* SA12) group into one of six phylogenetically distinct clades (named Clades I–VI), and (ii) half (12 strains) produce multiple AHLs, two strains produce two AHLs, four strains produce one AHL, and we were unable to detect AHL in six (nine in total of 57; see Table S4).

All representatives of Clade I produce 8–11 different AHLs, with 3-oxo-C6-HSL in ‘high’ quantities. *A. wodanis* SA12 is placed immediately outside of Clade I, but shows an AHL profile that resembles those of Clade I. In Clade II, *A. fischeri* ES114 was the only representative tested, and detectable levels of 5 AHLs were identified. This contrasts the earlier finding when using SWT medium where we found six AHLs (C4-HSL was below LOQ when grown in LB25). Clade III represents a group of *A. wodanis* strains that produce ‘medium’ amount of 3-OH-C10-HSL, except for *A. wodanis* 01/401, which also produces ‘medium’ amount of C8-HSL. Clade IV represents two *Aliivibrio* strains (*Aliivibrio* R8-63 and *Aliivibrio* R8-67) that are phylogenetically distinct from other Aliivibrio species and have no apparent AHL production. Several representatives from the *Vibrio* genus were grouped into Clade V and show somewhat different AHL profiles. Specifically, no AHLs were detected in two members, that is, *Vibrio* sp. B9-25 and *V. tapetis* 99/196. However, it should be noted that the B9-25 strain grew slowly and had only reached an OD of 0·1 at the time of measurement. *Vibrio splendidus* LMG 19031 was found to produce one AHL, that is, ‘high’ quantities of 3-OH-C4-HSL, *V. splendidus* 02/066 produced C4-HSL and 3-OH-C4-HSL, and the remaining two strains produced multiple AHLs. *Vibrio anguillarum* NB10 produced 3-OH-C6-HSL (‘medium’), 3-oxo-C10-HSL (‘medium’) and 3-OH-C10-HSL (‘low’). This is in overall agreement with earlier findings (Milton *et al*. 2001; Buchholtz *et al*. 2006). Photobacteria formed Clade VI, and none of these produced AHLs above LOQ. A complete list of quantitative measurements is shown in Table S4.

## Discussion

We have used a sensitive HPLC-MS/MS method and AHL standards to first detect (qualitative analysis) and then quantify (with an appropriate internal standard) the presence of fifteen different AHLs in 57 different *Vibrionaceae* strains, five, seven and 45 of which belong to the *Photobacterium*,*Vibrio* and *Aliivibrio* genera, respectively. AHL quantification was mapped onto a host phylogeny tree. Typically, closely related strains produced similar AHL profiles, but different profiles were also found among closely related strains (e.g. *Vibrio splendidus*).

### HPLC-MS/MS as a method to detect and quantify AHLs in bacterial cultures

Methods to detect AHLs in biological samples are becoming increasingly more sensitive, and different types of MS protocols have recently been developed with the purpose to find these signalling molecules in low concentrations, for example, picomoles of AHLs (Makemson *et al*. 2006). However, the use of such protocols for screening purposes has been hampered by several factors. MS methods typically suffer from the demand of relatively large sample sizes in order to detect low concentrations of AHLs. In addition, extraction and analysis procedures are time-consuming and relatively costly. Some protocols include solid-phase extraction to isolate AHLs (Fekete *et al*. 2010), and this further adds to the handling time. In this study, we set out to facilitate small sample sizes and minimum handling times, along with scalability and automated analysis of samples. In our extraction protocol, we used 75 μl spent growth media and ethyl acetate as the extraction solvent. AHLs in spent growth media were stabilized with hydrochloric acid before storage at −20°C and subsequent HPLC-MS/MS analysis. Samples could be safely stored for 2 months without affecting the quality of the HPLC-MS/MS analysis. The ethyl acetate extracts were dried and redissolved in 20% acetonitrile, which was run on HPLC-MS/MS. During LC, acetonitrile was used as the eluent for the reverse-phase chromatography. Methanol has been used in the majority of earlier work (Hanzelka *et al*. 1999; Yates *et al*. 2002), but it was recently shown that methanol forms more adducts compared to acetonitrile (Cataldi *et al*. 2008). The extracted AHLs were typically run in sets of 200 samples per day. MS/MS in SRM mode was regarded as the suitable method, based on previous reports (Morin *et al*. 2003; Gould *et al*. 2006; Ortori *et al*. 2011; May *et al*. 2012), because the SRM method provides better selectivity for C4 AHLs (3-OH-C4-HSL, C4-HSL, 3-oxo-C4-HSL) than full-scan Orbitrap. Our results also showed that C4-chained AHLs were more difficult to detect and quantify compared to the longer-chained AHLs. There are two main reasons for this. First, C4-chained AHLs are detected as relatively broad peaks near the injection peak where the mass baseline (noise level) is high (Fig. S1). Second, the C4-chained AHLs elute when the mobile-phase solvent consists mostly of water (approximately 90% water and 10% acetonitrile), which then typically also contain more metal ions such as sodium and potassium. The AHLs are, therefore, more likely to form, for example, sodium adducts, which create additional problems during the interpretations of collected data. Chances of running into problems with sodium and other unwanted adducts are, however, reduced by using acetonitrile and water as mobile-phase solvents (Cataldi *et al*. 2008).

Perhaps the most important limitation to experiments as described here is that the AHL production in bacteria is greatly dependent on growth parameters. Differences in, for example, medium composition, growth temperatures, starting optical densities and shaker speed can greatly affect the results. Hence, as we have experienced during this study, reproducing the growth conditions and results from previous studies can be very difficult. Moreover, different species and/or isolates can have very different growth requirement; hence, direct comparison of results from different species/isolates can also be challenging.

### AHL production in *Vibrionaceae*

In *Vibrionaceae,* the production of specific AHLs has been carefully studied for several model bacteria, for example, *Aliivibrio fischeri* (Kuo *et al*. 1994), *Vibrio anguillarum* (Buchholtz *et al*. 2006) and *Vibrio harveyi* (Cao and Meighen 1989), and their AHL-producing enzymes have been characterized in detail (Schaefer *et al*. 1996; Milton *et al*. 1997, 2001; Hanzelka *et al*. 1999). In *A. fischeri,* AinS is responsible for the production of C8-HSL (Hanzelka *et al*. 1999), whereas LuxI is responsible for the production of 3-oxo-C6-HSL and C6-HSL (Kuo *et al*. 1994). Our results suggest the existence of at least six AHLs in *A. fischeri* ES114. No previous study has reported this many AHLs in *A. fischeri*. One explanation for this could be that previous studies have used techniques, for example, field desorption (FD) ionization with double-focusing (magnetic and electric sectors in series) MS (Eberhard *et al*. 1981), desorption chemical ionization probe and electron impact ionization with magnetic sector MS (Kuo *et al*. 1994), that differ from our LTQ MS with SRM approach. AHL concentrations reported by Stabb *et al*. (2008) are based on a method described earlier (Gray and Greenberg 1992) where radioisotopes were used. Hence, they could detect very low levels of AHLs, but their analysis was restricted to specific AHLs (3-oxo-C6-HSL and C8-HSL). In our approach, we searched for 15 different AHLs by using their specific retention times during HPLC, their molecular mass to charge ratio and their fragmentation product (lactone ring; m/z 102·055). There is significantly more AHLs than has been searched for earlier, for example, Kuo *et al*. (Kuo *et al*. 1994) searched for 6 and May *et al*. (May *et al*. 2012) searched for 8 different AHLs. May *et al*., also searched for C4-HSL, but they used LM medium (LB media with 2% salt). In a similar medium (LB25), we also found C4-HSL below LOQ in *A. fischeri* ES114.

Although we have detailed knowledge on a few model bacteria, the distribution of AHL-based QS systems in environmental *Vibrionaceae* isolates has been unclear. It has also been largely unknown whether the majority of *Vibrionaceae* members produce similar or very different AHL profiles. This was recently addressed by two independent studies, in which they screened for QS signals in 25 (Yang *et al*. 2011) and 106 (García-Aljaro *et al*. 2012) *Vibrionaceae* strains. Using different biosensors and TLC, they found a variety of AHLs. Although these semi-quantitative methods are efficient methods for screening many samples for the presence of QS signal molecules, they are also inherently limited by (i) their abilities to discriminate between the many different AHLs (chain lengths, hydroxy/oxo modifications); (ii) their abilities to discriminate between AHLs and AHL mimics (chemically different molecules that have similar activities as AHLs in biosensor assays); and (iii) that the methods are typically less reliable when detecting AHLs at very low concentrations. In this study, we screened 57 *Vibrionaceae* members for AHL production using the HPLC-MS/MS and AHL standards and provided quantitative measurements of fifteen different AHLs (C4–C12-chained AHLs with unsubstituted 3′ carbons or 3′ Oxo or OH modifications; Fig. 1). Thirteen of the fifteen tested AHLs were found in the bacterial samples surveyed in this study, C12-HSL and 3-oxo-C12-HSL were never detected, and C10-HSL and 3-OH-C12-HSL were rarely found. As in previous studies, we conclude that AHLs are found widespread in *Vibrio* and *Aliivibrio*, with only a handful of isolates completely lacking AHL production (under our defined growth conditions, and when using our detection method). Furthermore, we show that closely related species typically produce similar AHL profiles, although interesting exceptions do exist.

### Why multiple AHLs in bacteria?

The phylogenetic tree in Fig. 2 shows that half of the tested strains produce multiple AHLs, whereas others produce one, two or even none. Likely explanations for this AHL diversity are for example, that one AHL can bind to and regulate the activity of different receivers or that several AHLs bind to and regulate same receiver(s). Both of these cases can be seen in *A. fischeri* where the AinS product C8-HSL binds to both AinR and LuxR at intermediate cell densities. In addition to binding C8-HSL, LuxR also binds 3-oxo-C6-HSL at high cell densities (Lupp *et al*. 2003). Moreover, LuxR type of regulators can be classified into five categories based on their activation mechanisms (Stevens *et al*. 2011). The first type (TraR type) is activated by tight AHL binding; the second type (LuxR type) represents dimers that are activated after AHL binding; the third type (MrtR type) represents nonfunctional monomers that dimerize and are activated upon AHL binding; the fourth type (EsaR type) represents functional dimers that are inactivated upon AHL binding, and finally the fifth type (SdiA type) represents monomers that are activated only upon binding to AHL, but still remain as monomers. This illustrates some of the diversity of activation mechanisms that AHLs are involved in.

## Supporting Information

**Figure S1** Ion chromatograms of AHLs with retention time (RT). **Figure S2** Linearity of AHL measurement over a broad concentration range. **Table S1** AHLs standards with their retention time and fragments generated by the SRM method used in this study for analysis. **Table S2** Validation of precision and accuracy of *Escherichia coli* ArcticExpress (DE3) supernatant spiked with AHLs. **Table S3** AHL concentrations in spent media from *A. fischeri* ES114. Measurements (average of three technical replicates with standard deviation) from two biological replicates are shown. **Table S4** Quantitative analysis of fifty-seven bacteria from *Vibrionaceae* family. **Dataset S1** 16S rRNA gene sequences from 45 strains used to make the phylogenetic tree in Fig. 2.
