# Supplementary material for: Presence of acyl-homoserine lactones in 57 members of the Vibrionaceae family
Source: J Appl Microbiol. 2013 Jun 28;115(3):835–47. doi: 10.1111/jam.12264 (PMC3910146; doi:10.1111/jam.12264)
Supplement: Supplementary file 1 — Figure S1 Ion chromatograms of AHLs with retention time (RT). Figure S2 Linearity of AHL measurement over a broad concentration range. Table S1 AHLs standards with their retention time and fragments generated by the SRM method used in this study for analysis. Table S2 Validation of precision and accuracy of Escherichia coli ArcticExpress (DE3) supernatant spiked with AHLs. Table S3 AHL concentrations in spent media from A. fischeri ES114. Measurements (average of three technical replicates with standard deviation) from two biological replicates are shown. Table S4 Quantitative analysis of fifty-seven bacteria from Vibrionaceae family. Dataset S1 16S rRNA gene sequences from 45 strains used to make the phylogenetic tree in Fig. 2. [file jam0115-0835-sd1.docx]

**Supporting information**

**
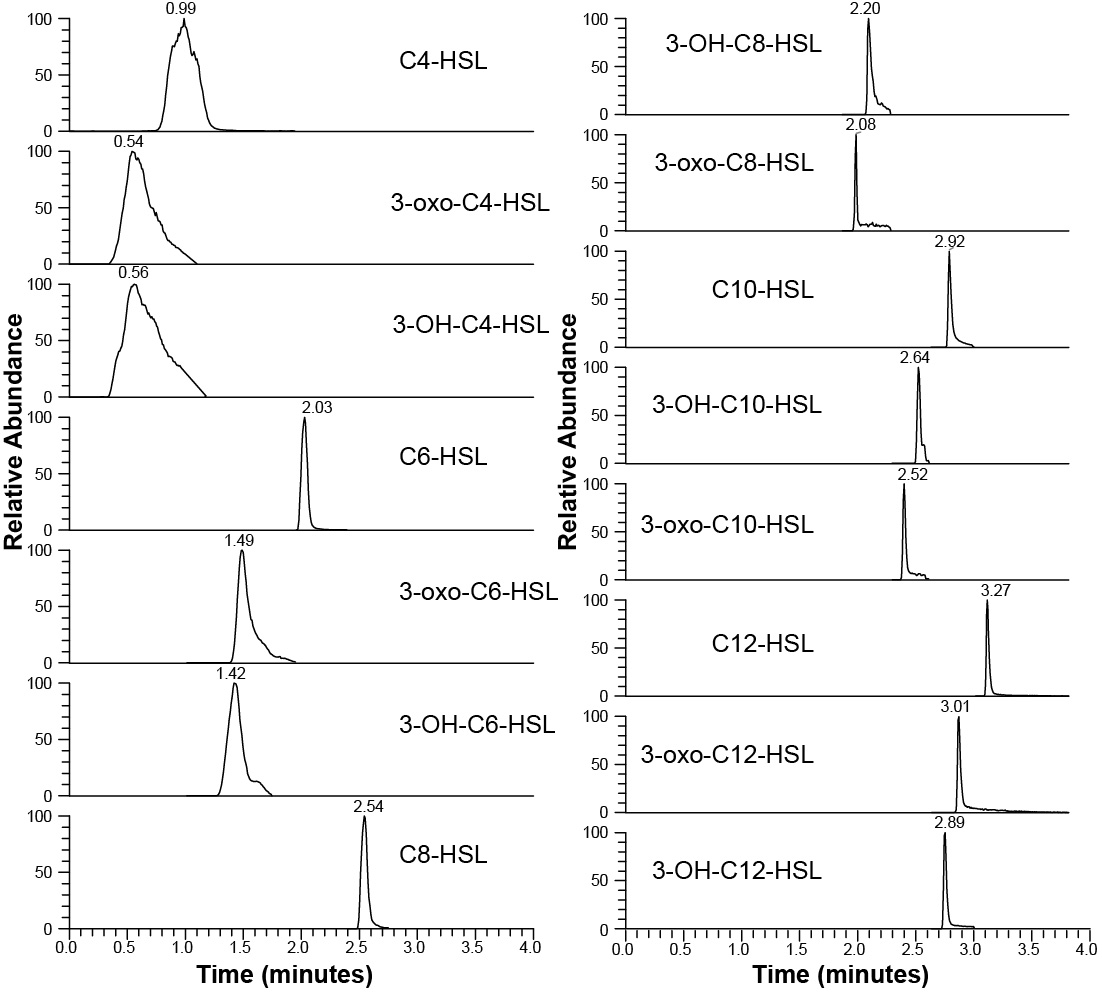
**

**Figure S1:** Ion chromatograms of AHLs with retention time. Relative abundance (in percentage) of particular ions are shown against retention time (in minutes). Plots are generated using the quant Browser algorithm in the Thermo Xcalibur MS analysis pakage. Retention time ranges are given in Table S1.

**
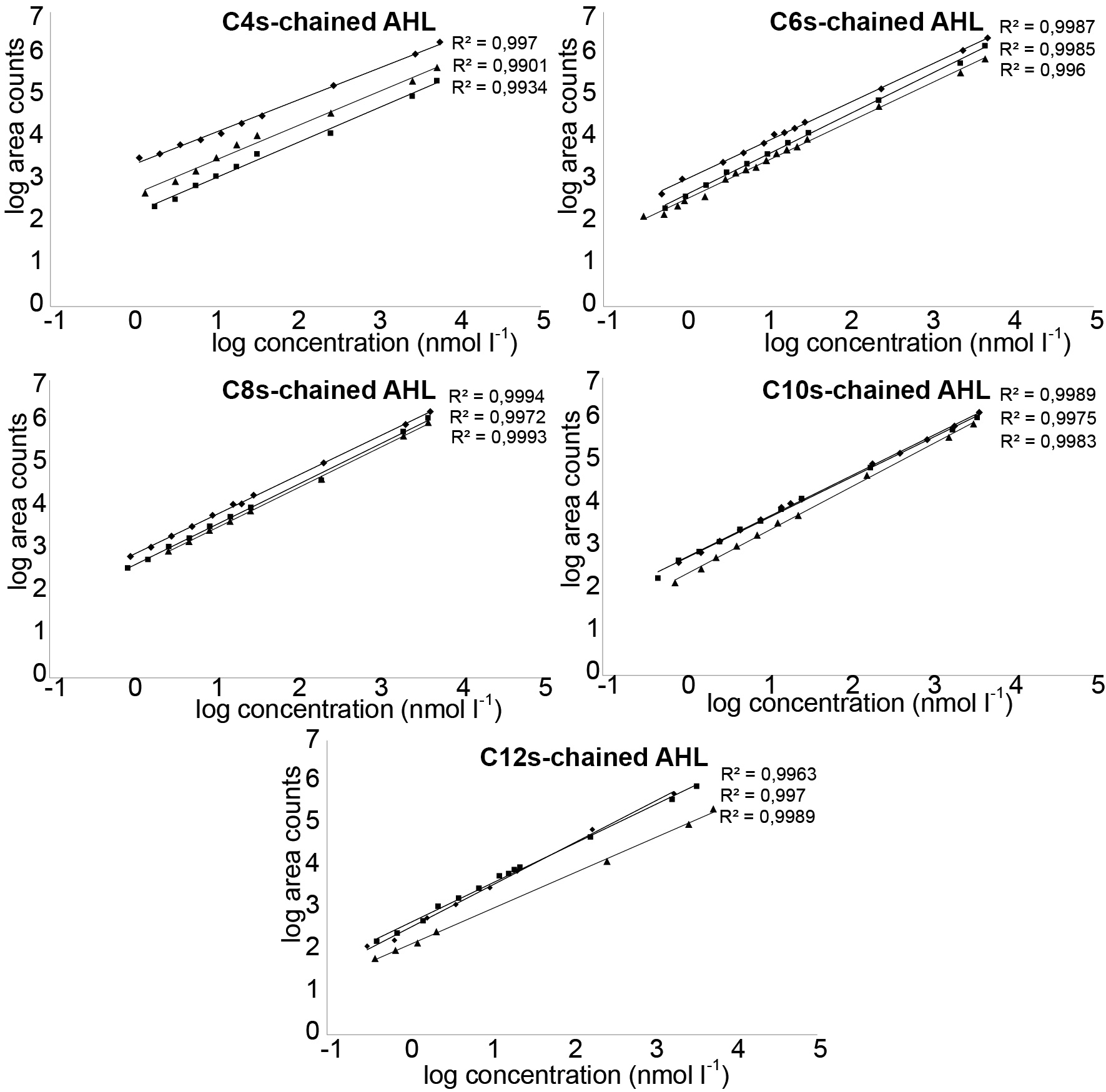
**

**Figure S2:** Linearity of AHL measurement over a broad concentration range. The standard AHL mix was diluted in LB25 before extraction and subsequent analysis on HPLC-MS/MS. (■) 3-OH, (⯅) 3-Oxo, (⯁) unsubstituted.

**Table S1:** AHL standards with their retention time and fragments generated by the SRM method used in this study.

| **AHL**^‡^ | **Molecular**  **Formula** | **Parent ion***  **[M + H]^+^**  **(m/z)** | **Fragment ion**  **(m/z)** | **Range of RT**^†^  **(min)** | **SRM**^§^  **Segments and scan events** |
| --- | --- | --- | --- | --- | --- |
| C4-HSL | C_8_H_13_NO_3_ | 172·09682 | 102·055 | 0-1·95 | 1·1 and 2·1 |
| 3-oxo-C4-HSL | C_8_H_11_NO_4_ | 186·07608 | 102·055 | 0-1 | 1·2 |
| 3-OH-C4-HSL | C_8_H_13_NO_4_ | 188·09173 | 102·055 | 0-1 | 1·3 |
| 3-OH-C6-HSL | C_10_H_17_NO_4_ | 216·12303 | 102·055 | 1-1·95 | 2·2 |
| 3-oxo-C6-HSL | C_10_H_15_NO_4_ | 214·10738 | 102·055 | 1-1·95 | 2·3 |
| C6-HSL | C_10_H_17_NO_3_ | 200·12812 | 102·055 | 1·95-2·4 | 3·1 |
| 3-oxo-C8-HSL | C_12_H_19_NO_4_ | 242·13868 | 102·055 | 1·95-2·4 | 3·2 |
| 3-OH-C8-HSL | C_12_H_21_NO_4_ | 244·15433 | 102·055 | 1·95-2·4 | 3·3 |
| C8-HSL | C_12_H_21_NO_3_ | 228·15942 | 102·055 | 2·4-2·75 | 4·1 |
| 3-oxo-C10-HSL | C_14_H_23_NO_4_ | 270·16998 | 102·055 | 2·4-2·75 | 4·2 |
| 3-OH-C10-HSL | C_14_H_25_NO_4_ | 272·18563 | 102·055 | 2·4-2·75 | 4·3 |
| C10-HSL | C_14_H_25_NO_3_ | 256·19072 | 102·055 | 2·75-3·15 | 5·1 |
| 3-OH-C12-HSL | C_16_H_29_NO_4_ | 300·21693 | 102·055 | 2·75-3·15 | 5·2 |
| 3-oxo-C12-HSL | C_16_H_27_NO_4_ | 298·20128 | 102·055 | 2·75-4·0 | 5·3 and 6·1 |
| C12-HSL | C_16_H_29_NO_3_ | 284·22202 | 102·055 | 3·15-4·0 | 6·2 |

^‡^ AHL, Acyl homoserine lactones

* Monoisotopic mass values were calculated using Xcalibur software package (2·07 Thermo Scientific).

^†^ Range of the RT, Retention Time on which the segment and events were applied.

^§^ SRM, Single reaction monitoring method which is divided into segments and scan events on specific retention time

**Table S2:** Validation of precision and accuracy of *Escherichia coli* ArcticExpress (DE3) supernatant spiked with AHLs. Cells were grown overnight at 30°C and a known amount AHL standards was added to the harvested supernatant of used medium. Samples were subjected to extraction and tested on HPLC-MS/MS. C10-HSL was used as internal standard.

| **AHLs** | **AHLs^*^ [nmol l^-1^]** | **Average detected**  **AHLs^**^ [nmol l^-1^]** | **Accuracy**  **(%) deviation**^†^ | **Precision**  **(RSD%)**^††^ |
| --- | --- | --- | --- | --- |
| **C4-HSL** | 31·4 | 42·4 | 35·2 % | 4·9 % |
|  | 1817·4 | 1781·0 | -2·0 % | 1·6 % |
|  | 2726·1 | 2509·7 | -7·9 % | 3·1 % |
| **3-OHC4-HSL** | 27·1 | 29·1 | 7·6 % | 5·3 % |
|  | 1567·9 | 1410·0 | -10·1 % | 10·1 % |
|  | 2351·9 | 2027·8 | -13·8 % | 8·6 % |
| **3-oxo-C4-HSL** | 27·4 | 29·0 | 5·9 % | 13·0 % |
|  | 1585·0 | 1482·0 | -6·5 % | 9·6 % |
|  | 2377·5 | 2199·6 | -7·5 % | 5·6 % |
| **C6-HSL** | 23·5 | 24·1 | 2·3 % | 3·0 % |
|  | 1362·7 | 1466·2 | 7·6 % | 0·5 % |
|  | 2044·0 | 1909·3 | -6·6 % | 3·3 % |
| **3-oxo-C6-HSL** | 25·1 | 24·9 | -0·9 % | 12·7 % |
|  | 1455·7 | 1368·7 | -6·0 % | 2·6 % |
|  | 2183·5 | 1860·8 | -14·8 % | 1·6 % |
| **3-OH-C6-HSL** | 25·9 | 22·6 | -12·7 % | 8·6 % |
|  | 1500·0 | 1493·4 | -0·4 % | 2·7 % |
|  | 2250·0 | 2173·0 | -3·4 % | 2·7 % |
| **C8-HSL** | 24·8 | 24·5 | -1·5 % | 8·8 % |
|  | 1437·3 | 1377·2 | -4·2 % | 3·2 % |
|  | 2155·9 | 1991·8 | -7·6 % | 1·0 % |
| **3-oxo-C8-HSL** | 22·8 | 22·9 | 0·5 % | 0·1 % |
|  | 1318·1 | 1396·6 | 6·0 % | 7·2 % |
|  | 1977·1 | 1941·2 | -1·8 % | 9·3 % |
| **3-OH-C8-HSL** | 22·9 | 16·0 | -30·0 % | 11·3 % |
|  | 1327·1 | 1458·5 | 9·9 % | 6·5 % |
|  | 1990·6 | 2154·8 | 8·3 % | 2·1 % |
| **3-oxo-C10-HSL** | 19·6 | 19·3 | -1·6 % | 1·6 % |
|  | 1133·4 | 1147·3 | 1·2 % | 2·7 % |
|  | 1700·1 | 1658·9 | -2·4 % | 2·6 % |
| **3-OH-C10-HSL** | 21·5 | 23·5 | 9·5 % | 5·0 % |
|  | 1244·0 | 1284·4 | 3·3 % | 4·5 % |
|  | 1865·9 | 1831·0 | -1·9 % | 2·2 % |
| **C12-HSL** | 20·8 | 19·2 | -7·4 % | 3·1 % |
|  | 1201·5 | 1281·9 | 6·7 % | 4·4 % |
|  | 1802·2 | 1815·3 | 0·7 % | 3·8 % |
| **3-OH-C12-HSL** | 18·5 | 17·8 | -3·8 % | 3·4 % |
|  | 1068·6 | 1080·0 | 1·1 % | 3·4 % |
|  | 1602·9 | 1503·5 | -6·2 % | 2·7 % |
| **3-oxo-C12-HSL** | 15·9 | 16·1 | 1·4 % | 9·0 % |
|  | 917·9 | 943·5 | 2·8 % | 8·7 % |
|  | 1376·9 | 1303·7 | -5·3 % | 7·9 % |

**^*^** Amount of AHL added (or spiked) into culture supernatant of *Escherichia coli* ArcticExpress (DE3) grown overnight at 30°C in LB1% medium

**^**^** Average AHL concentration from three technical replicates detected by LC-MS. The values were quantified using C10-HSL as an internal standard.

^†^ Accuracy is the percentage deviation from the true value.

^††^ Precision is the relative standard deviation (RSD).

**Table S3:** AHL concentrations in spent media from *A. fischeri* ES114. Measurements (average of three technical replicates with standard deviation) from two biological replicates are shown.

|  | **Sample** |  | **C4-HSL** | **3Oxo-C6-HSL** | | **C6-HSL** | **3OH-C8-HSL** | **C8-HSL** | **C10-HSL** |
| --- | --- | --- | --- | --- | --- | --- | --- | --- | --- |
| **Biological**  **Replicate** | **OD_60_** | **Temp** | **nM** | **nM** | | **nM** | **nM** | **nM** | **nM** |
| 1 | 0.46 | **12°C** | 61±3 | 566±25 | | 19.0±1.3 | 1.1±0.2 | 63±4 | 1.7±0.4 |
|  | 1.04 |  | 79±4 | 1280±240 | | 65±5 | 6.6±1.3 | 713±38 | 15±1 |
|  | 0.98 |  | 80±5 | 1400±230 | | 73±5 | 6.4±0.2 | 812±35 | 17±2 |
|  |  |  |  |  | |  |  |  |  |
| 2 | 0.52 |  | 63±1 | 780±46 | | 26.0±0.8 | 1.6±0.2 | 97±3 | 2.6±0.2 |
|  | 1.02 |  | 80±5 | 1300±500 | | 78±5 | 5.0±0.9 | 770±60 | 17±2 |
|  | 1.02 |  | 78±3 | 1400±400 | | 77±4 | 5.35±0.70 | 800±70 | 16±1 |
|  |  |  |  |  | |  |  |  |  |
| 1 | 0.66 | **22°C** | 69±2 | | 20.6±0.3 | 2.8±0.2 | 0.92±0.10 | 54±2 | 1.5±0.1 |
|  | 1.6 |  | 82±4 | | 108±70 | 12.0±0.8 | 5.0±0.6 | 228±7 | 5.3±0.4 |
|  | 2.44 |  | 95±10 | | 210±120 | 33.8±0.9 | 14.8±1.1 | 637±14 | 13.5±0.9 |
|  | 2.96 |  | 138±19 | | 210±70 | 101±6 | 25±3 | 2166±37 | 47.5±5.2 |
|  |  |  |  | |  |  |  |  |  |
| 2 | 0.66 |  | 67.9±2.7 | | 20±5 | 2.7±0.3 | 1.1±0.1 | 51±4 | 1.5±0.3 |
|  | 1.7 |  | 80±2 | | 92±17 | 11.8±0.7 | 5.2±0.5 | 257±9 | 5.1±0.2 |
|  | 2.64 |  | 98±7 | | 168±16 | 35±3 | 13±3 | 851±40 | 17.8±0.6 |
|  | 2.34 |  | 119±15 | | 75±56 | 62±9 | 18.6±0.9 | 1745±125 | 39±6 |
|  |  |  |  |  | |  |  |  |  |
| 1 | 1.1 | **30°C** | 69±10 | -- | | 2.6±0.3 | 1.0±0.1 | 87±5 | 2.20±0.12 |
|  | 1.4 |  | 70±2 | -- | | 4.8±0.1 | 1.3±0.1 | 148±6 | 3.8±0.1 |
|  | 1.58 |  | 64±8 | -- | | 4.9±0.2 | 1.4±0.5 | 155±11 | 3.9±0.1 |
|  | 1.48 |  | 84±5 | 0.19±0.17 | | 5.9±0.7 | 1.2±0.1 | 172±7 | 4.7±0.5 |
|  |  |  |  |  | |  |  |  |  |
| 2 | 1.24 |  | 61±4 | 0.22±0.06 | | 2.8±0.1 | 0.9±0.2 | 96±2 | 2.5±0.3 |
|  | 1.4 |  | 67±3 | 0.14±0.20 | | 4.4±0.4 | 1.1±0.1 | 143±10 | 3.2±0.3 |
|  | 1.54 |  | 76±2 | 0.16±0.14 | | 4.7±0.2 | 1.3±0.1 | 167±3 | 4.3±0.5 |
|  | 1.36 |  | 85±2 | 0.15±0.07 | | 5.2±0.5 | 1.2±0.1 | 172±7 | 4.2±0.2 |

The tandem MS SRM method was used for quantitative analysis with internal standard (3-oxo-C12-HSL). Values are given as nmol l^-1^ of AHLs calculated against one point calibration of known standards. Two parallel colonies were grown and harvested at different optical densities.

Tested samples were grown at 12, 22 and 30°C in SWT medium. Cultures were grown from OD_600_ 0·05. --, values below LOQ.

**Table S4:** Quantitative analysis of fifty seven bacteria from the *Vibrionaceae* family. Concentrations of AHLs are grouped into four categories: “High” >5 µmol l^-1^ (orange), “medium” 25-5000 nmol l^-1^ (blue), “low” <25 nmol l^-1^ (yellow), and below LOQ (--). IS, internal standard. *, data is based on one biological replicate (instead of two).

|  |  |  | **3-OH-C4-HSL** | **3-oxo-C4-HSL** | **C4-HSL** | **3-OH-C6-HSL** | **3-oxo-C6-HSL** | **C6-HSL** | **3-OH-C8-HSL** | **3-oxo-C8-HSL** | **C8-HSL** | **3-OH-C10-HSL** | **3-oxo-C10-HSL** | **C10-HSL** | **3-OH-C12-HSL** | **3-oxo-C12-HSL** | **C12-HSL** |
| --- | --- | --- | --- | --- | --- | --- | --- | --- | --- | --- | --- | --- | --- | --- | --- | --- | --- |
| **Clade;**  **species** | **Strain** | **OD_600_** | **nmol l^-1^** | **nmol l^-1^** | **nmol l^-1^** | **nmol l^-1^** | **nmol l^-1^** | **nmol l^-1^** | **nmol l^-1^** | **nmol l^-1^** | **nmol l^-1^** | **nmol l^-1^** | **nmol l^-1^** | **nmol l^-1^** | **nmol l^-1^** | **nmol l^-1^** | **nmol l^-1^** |
| No clade; *A.wodanis* | SA12 | 1•3±0•0 | -- | 16±1 | -- | -- | 2000±1000 | 2200±400 | -- | 1600±200 | 200±40 | 500±100 | 70±10 | 9±3 | 7•6±4•5 | -- | IS |
| Clade I;  *A.logei* | 29985 | 1•9±0•0 | 20±7 | 190±60 | 140±30 | 130±50 | 50000±8000 | 1800±300 | 10±3 | 1700±300 | 50±6 | 160±30 | 40±10 | -- | -- | -- | IS |
| *A.logei* | SES -5 | 1•15±0•5 | -- | 180±60 | 140±30 | 140±30 | 43000±9000 | 1400±400 | 20±6 | 1600±400 | 40±10 | 120±10 | 60±10 | -- | -- | -- | IS |
| *A.logei* | SES03-1 | 1•5* | -- | 820±130 | 300±10 | 110±5 | 50000±1600 | 3400±200 | 80±3 | 3000±300 | 160±5 | 640±40 | 90±20 | 8•0±0•5 | -- | -- | IS |
| *A.logei* | 90/1667 | 0•85±0•05 | -- | 70±20 | 100±30 | 70±20 | 23000±5000 | 900±300 | -- | 900±200 | 30±10 | 60±4 | 30±6 | -- | -- | -- | IS |
| *A.salmonicida* | LFl1238 | 2•15±0•05 | -- | 50±10 | 100±20 | -- | 12000±1300 | 700±100 | -- | 580±50 | 50±2 | 110±4 | 60±5 | -- | IS | -- | -- |
| *A.salmonicida* | 43839 | 1•25±0•05 | -- | 120±20 | 180±20 | -- | 21000±3000 | 1400±100 | -- | 900±80 | 70±4 | 100±4 | 80±6 | 3•1±2•4 | -- | -- | IS |
| *A.sp.* | R5-43 | 1•2±0•6 | -- | 80±40 | 120±50 | 80±30 | 27000±9000 | 1400±500 | 10±6 | 1100±300 | 50±20 | 100±30 | 30±10 | -- | 1•9±0•2 | -- | IS |
| *A.sp* | R5-42 | 1•15±0•05 | -- | 120±20 | 170±10 | 100±10 | 31000±5000 | 1200±100 | 12±1 | 1300±60 | 60±3 | 100±6 | 50±3 | -- | 2•0±0•2 | -- | IS |
| *A.sp* | R8-70 | 1•35±0•05 | -- | 55±30 | 90±10 | 50±8 | 15000±2000 | 600±40 | -- | 600±70 | 40±6 | 190±10 | 50±7 | -- | -- | -- | IS |
| *A.sp* | R8-66 | 1•3±0•0 | -- | 30±6 | 50±5 | 30±3 | 8000±700 | 500±40 | -- | 600±40 | 20±2 | 150±6 | 50±5 | -- | -- | -- | IS |
| *A.sp* | R8-65 | 1•35±0•05 | -- | 60±20 | 90±8 | 50±7 | 15000±2000 | 700±30 | -- | 700±30 | 30±3 | 170±10 | 60±6 | -- | -- | -- | IS |
| *A.sp* | R8-61 | 1•3±0•0 | -- | 60±10 | 80±10 | 50±7 | 14000±1000 | 700±20 | -- | 800±40 | 30±2 | 190±6 | 70±4 | -- | -- | -- | IS |
| *A.sp* | R8-69 | 1•3±0•0 | -- | 30±10 | 150±15 | 25±5 | 7000±1300 | 500±70 | -- | 500±20 | 20±2 | 140±6 | 50±3 | -- | -- | -- | IS |
| *A.sp* | R8-68 | 1•3±0•0 | -- | 70±20 | 90±4 | 50±7 | 17000±2000 | 700±30 | -- | 760±30 | 30±1 | 180±7 | 60±5 | -- | -- | -- | IS |
| *A.sp* | B8-7 | 1•3±0•0 | -- | 50±20 | 80±9 | 50±4 | 15000±700 | 700±40 | -- | 740±25 | 30±1 | 160±10 | 60±6 | -- | -- | -- | IS |
| *A.sp* | B9-15 | 1•85±0•05 | -- | 180±10 | 200±15 | 110±7 | 41000±3000 | 2600±100 | -- | 1700±60 | 85±5 | 30±2 | 70±6 | -- | 2•2±0•1 | -- | IS |
| *A.sp* | R8-64 | 1•2±0•0 | -- | 30±5 | 40±8 | 20±2 | 6000±900 | 370±5 | -- | 360±20 | 20±1 | 60±3 | 30±1 | -- | -- | -- | IS |
| *A.sp* | MR17-66 | 0•45±0•05 | -- | 50±3 | 60±3 | 40±4 | 15000±900 | 500±30 | -- | 570±30 | 20±1 | 60±2 | 30±3 | -- | -- | -- | IS |
| *A.sp* | MR17-80 | 1•2±0•1 | -- | 50±20 | 80±10 | 50±8 | 18000±1600 | 640±60 | -- | 700±70 | 100±10 | 7±1 | 30±3 | -- | 10±1 | -- | IS |
| *A.sp* | MR17-69 | 1•3±0•0 | -- | 70±5 | 50±3 | 50±3 | 19000±600 | 600±50 | -- | 860±30 | 30±1 | 40±1 | 40±2 | -- | -- | -- | IS |
| *A.sp* | MR17-34 | 1•15±0•05 | -- | 100±5 | 70±6 | 65±4 | 26000±2000 | 1000±80 | -- | 1300±60 | 120±4 | 20±1 | 40±3 | -- | 3•3±0•1 | -- | IS |
| *A.sp* | MR17-70 | 1•15±0•05 | -- | 70±17 | 60±10 | 45±10 | 16000±2000 | 470±90 | -- | 640±100 | 24±10 | 30±4 | 30±5 | -- | -- | -- | IS |
| *A.sp* | MR17-77 | 1•2±0•1 | -- | 70±25 | 100±10 | 70±10 | 21000±1900 | 780±60 | -- | 770±70 | 120±5 | 22±18 | 30±3 | -- | 12•0±1•8 | -- | IS |
| *A.wodanis* | SR6 | 1•25±0•05 | -- | 15±5 | -- | 20±10 | 2400±600 | 100±30 | 11±7 | 80±10 | 120±20 | 240±30 | 60±7 | -- | 10±4 | -- | IS |
| Clade II;  *A.fischeri* | ES114 | 1•15±0•05 | -- | -- | -- | -- | 20±5 | 16 ±2 | 4•7±0•8 | -- | 430±30 | -- | -- | 9±1 | -- | IS | -- |
| Clade III;  *A.wodanis* | 89/5532 | 0•6±0•0 | -- | -- | -- | -- | -- | -- | -- | -- | -- | 40±2 | -- | IS | -- | -- | -- |
| *A.wodanis* | 90/325 | 0•6±0•0 | -- | -- | -- | -- | -- | -- | -- | -- | -- | 40±3 | -- | IS | -- | -- | -- |
| *A.wodanis* | 02/569 | 0•65±0•05 | -- | -- | -- | -- | -- | -- | -- | -- | -- | 60±4 | -- | IS | -- | -- | -- |
| *A.wodanis* | 02/382 | 0•9±0•0 | -- | -- | -- | -- | -- | -- | -- | -- | -- | 60±2 | -- | IS | -- | -- | -- |
| *A.wodanis* | 01/401 | 0•7±0•0 | -- | -- | -- | -- | -- | -- | -- | -- | 30±1 | 70±3 | -- | IS | -- | -- | -- |
| *A.wodanis* | 88/441T | 0•75±0•05 | -- | -- | -- | -- | -- | -- | -- | -- | -- | 220±70 | -- | -- | IS | -- | -- |
| *A.wodanis* | 06/194-A | 0•6±0•0 | -- | -- | -- | -- | -- | -- | -- | -- | -- | 160±10 | -- | IS | -- | -- | -- |
| *A.wodanis* | 06/194-B | 1•0±0•0 | -- | -- | -- | -- | -- | -- | -- | -- | -- | 230±20 | -- | IS | -- | -- | -- |
| *A.wodanis* | 06/139 | 1•1±0•1 | -- | -- | -- | -- | -- | -- | -- | -- | -- | 70±3 | -- | IS | -- | -- | -- |
| *A.wodanis* | 06/170 | 1•2±0•0 | -- | -- | -- | -- | -- | -- | -- | -- | -- | 140±10 | -- | -- | IS | -- | -- |
| *A.wodanis* | 06/178 | 1•4±0•1 | -- | -- | -- | -- | -- | -- | -- | -- | -- | 220±90 | -- | -- | IS | -- | -- |
| *A.wodanis* | 96/688 | 0•85±0•2 | -- | -- | -- | -- | -- | -- | -- | -- | -- | 50±10 | -- | IS | -- | -- | -- |
| *A.wodanis* | 04/17347 | 1•1±0•1 | -- | -- | -- | -- | -- | -- | -- | -- | -- | 40±1 | -- | IS | -- | -- | -- |
| *A.wodanis* | 03/160 | 1•4±0•0 | -- | -- | -- | -- | -- | -- | -- | -- | -- | 120±10 | -- | IS | -- | -- | -- |
| *A.wodanis* | BAA104 | 0•8±0•0 | -- | -- | -- | -- | -- | -- | -- | -- | -- | 170±10 | -- | -- | IS | -- | -- |
| Clade IV;  *A.sp.* | R8-63 | 1•6±0•0 | -- | -- | -- | -- | -- | -- | -- | -- | -- | -- | -- | -- | -- | -- | IS |
| *A.sp* | R8-67 | 1•65±0•05 | -- | -- | -- | -- | -- | -- | -- | -- | -- | -- | -- | -- | -- | -- | IS |
| Clade V;  *V.anguillarum* | NB10 | 1•1±0•0 | -- | -- | -- | 37±6 | -- | -- | -- | -- | -- | 6•2±0•6 | 80±6 | -- | IS | -- | -- |
| *V.sp.* | B9-25 | 0•1±0•0 | -- | -- | -- | -- | -- | -- | -- | -- | -- | -- | -- | -- | -- | -- | IS |
| *V.splendidus* | LMG19031 | 1•85±0•35 | 9400±1600 | -- | -- | -- | -- | -- | -- | -- | -- | -- | -- | IS | -- | -- | -- |
| *V.splendidus* | 02/14916 | 1•1±0•1 | 60±20 | -- | -- | 1570±300 | -- | -- | 11±3 | -- | -- | -- | -- | -- | IS | -- | -- |
| *V.splendidus* | 00/860 | 0•9±0•0 | 3000±400 | -- | -- | -- | -- | -- | -- | -- | -- | -- | -- | -- | -- | -- | IS |
| *V.splendidus* | 02/066 | 0•7±0•0 | 1400±140 | -- | 60±20 | -- | -- | -- | -- | -- | -- | -- | -- | -- | -- | -- | IS |
| *V.splendidus* | 04/276 | 0•8±0•0 | 85±15 | -- | 200±50 | 2500±300 | -- | 5±1 | 16±2 | -- | -- | -- | -- | IS | -- | -- | -- |
| *V.splendidus* | 03/122 | 2•1±0•0 | 6600±2200 | -- | -- | -- | -- | -- | -- | -- | -- | -- | -- | IS | -- | -- | -- |
| *V.tapetis* | 99/196 | 1•1±0•0 | -- | -- | -- | -- | -- | -- | -- | -- | -- | -- | -- | -- | IS | -- | -- |
| Clade VI;  *P.phosphoreum* | SP001 | 0•45±0•05 | -- | -- | -- | -- | -- | -- | -- | -- | -- | -- | -- | IS | -- | -- | -- |
| *P.phosphoreum* | SP002 | 1•15±0•05 | -- | -- | -- | -- | -- | -- | -- | -- | -- | -- | -- | IS | -- | -- | -- |
| *P.phosphoreum* | SP004 | 1•35±0•05 | -- | -- | -- | -- | -- | -- | -- | -- | -- | -- | -- | IS | -- | -- | -- |
| *P.phosphoreum* | SP005 | 1•1±0•1 | -- | -- | -- | -- | -- | -- | -- | -- | -- | -- | -- | IS | -- | -- | -- |
| *P.sp.* | SP044 | 0•1±0•0 | -- | -- | -- | -- | -- | -- | -- | -- | -- | -- | -- | IS | -- | -- | -- |

**Dataset S1**: 16S rRNA gene sequences for the 45 strains which are used to make the phylogenetic tree.

Asal=*A. salmonicida*; Asp=*A. sp*; Vsp=*V. sp*; Aw=*A. wodanis*; Al=*A. logei*; Vt=*V. tapetis*; Vspl=*V. splendidus*; Vang=*V. anguillarum*; Ghol=*G. hollisae*; Vcho=*V. cholera*; Plum=*P. luminescens*; Plei=*P. leiognathi*; Ppho=*P. phosphoreum*; Pang=*P. angustum*; Afis= *A. fischeri*; Asif= *A. sifiae*; Atho= *A. thorii*; Psp=*P. sp*; ?=missing data

10 20 30 40 50 60 70

....|....| ....|....| ....|....| ....|....| ....|....| ....|....| ....|....|

**AsalATCC43** **GCATGCAGGT** **GGTTCATTAA** **GTCAGATGTG** **AAAGCCCGGG** **GCTCAACCTC** **GGAACCGCAT** **TTGAAACTGG**

**AsalLFl123** **GCATGCAGGT** **GGTTCATTAA** **GTCAGATGTG** **AAAGCCCGGG** **GCTCAACCTC** **GGAACCGCAT** **TTGAAACTGG**

**AspR863**  **GCATGCAGGT** **GGTTCATTAA** **GTCAGATGTG** **AAAGCCCGGG** **GCTCANCCTC** **GGAACCGCAT** **TTGAAACTGG**

**AspR867**  **GCATGCAGGT** **GGTTCATTAA** **GTCAGATGTG** **AAAGCCCGGG** **GCTCNNCCTC** **GGAACCGCAT** **TTGAAACTGG**

**VspB925**  **GCATGCAGGT** **GGTTTGTTAA** **GTCAGATGTG** **AAAGCCCGGG** **GCTNNNCCCC** **GGAAGGTCAT** **TTGAAACTGG**

**AspB915**  **GCATGCAGGT** **GGTTCATTAA** **GTCAGATGTG** **AAAGCCCGGG** **GCTCAACCTC** **GGAACCGCAT** **TTGAAACTGG**

**AwSA12**  **??????????** **GGTTCATTAA** **GTCAGATGTG** **AAAGCCCGGG** **GCTCAACCTC** **GGAACCGCAT** **TTGAAACTGG**

**AspMR1766**  **GCATGCAGGT** **GGTTCATTAA** **GTCAGATGTG** **AAAGCCCGGG** **GCTCAACCTC** **GGAACCGCAT** **TTGAAACTGG**

**AwATCC1538** **GCATGCAGGT** **GGTTCATTAA** **GTCAGATGTG** **AAAGCCCGGG** **GCTCAACCTC** **GGAACCGCAT** **TTGAAACTGG**

**Aw02569**  **GCATGCAGGT** **GGTTTGTTAA** **GTCAGATGTG** **AAAGCCCGGG** **GCTCAACCTC** **GGAACCGCAT** **TTGAAACTGG**

**Aw01401**  **GCATGCAGGT** **GGTTNGTTAA** **GTCAGATGTG** **AAAGCCCGGG** **GCTCAACCTC** **GGAACCGCAT** **TTGAAACTGG**

**Aw06139A**  **GCATGCAGGT** **GGTTTGTTAA** **GTCAGATGTG** **AAAGCCCGGG** **GCTCAACCTC** **GGAACCGCAT** **TTGAAACTGG**

**Al901667**  **GCATGCAGGT** **GGTTCATTAA** **GTCAGATGTG** **AAAGCCCGGG** **GCTCAACCTC** **GGAACCGCAT** **TTGAAACTGG**

**Aw03160**  **GCATGCAGGT** **GGTTTGTTAA** **GTCAGATGTG** **AAAGCCCGGG** **GCTCAACCTC** **GGAACCGCAT** **TTGAAACTGG**

**AwSR6**  **GCATGCAGGT** **GGTTCATTAA** **GTCAGATGTG** **AAAGCCCGGG** **GCTCAACCTC** **GGAACCGCAT** **TTGAAACTGG**

**Vt99196**  **GCATGCAGGT** **GGTTCGTTAA** **GTCAGATGTG** **AAAGCCCGGG** **GCTCAACCTC** **GGAACTGCAT** **TTGAAACTGG**

**VsplLMG190** **GCATGCAGGT** **GGTTCATTAA** **GTCAGATGTG** **AAAGCCCGGG** **GCTCAACCTC** **GGAACTGCAT** **TTGAAACTGG**

**Vspl02066**  **GCATGCAGGT** **GGTTCATTAA** **GTCAGATGTG** **AAAGCCCGGG** **GCTCAACCTC** **GGAACTGCAT** **TTGAAACTGG**

**Vspl04276**  **GCATGCAGGT** **GGTTCATTAA** **GTCAGATGTG** **AAAGCCCGGG** **GCTCAACCTC** **GGAACTGCAT** **TTGAAACTGG**

**Vspl021491** **GCATGCAGGT** **GGTTCATTAA** **GTCAGATGTG** **AAAGCCCGGG** **GCTCAACCTC** **GGAACTGCAT** **TTGAAACTGG**

**VangNB10**  **GCATGCAGGT** **GGTGGATTAA** **GTCAGATGTG** **AAAGCCCGGG** **GCTCAACCTC** **GGAACCGCAT** **TTGAAACTGG**

**GholLMG177** **GCATGCAGGC** **GGTCTGTTAA** **GCAAGATGTG** **AAAGCCCCGG** **GCTTAACCTG** **GGAGTGGCAT** **TTTGAACTGG**

**VchoATCC14** **GCATGCAGGT** **GGTTTGTTAA** **GTCAGATGTG** **AAAGCCCTGG** **GCTCAACCTA** **GGAATCGCAT** **TTGAAACTGA**

**VchoO1clas** **GCATGCAGGT** **GGTTTGTTAA** **GTCAGATGTG** **AAAGCCCTGG** **GCTCAACCTA** **GGAATCGCAT** **TTGAAACTGA**

**VchoO1E1To** **GCATGCAGGT** **GGTTTGTTAA** **GTCAGATGTG** **AAAGCCCTGG** **GCTCAACCTA** **GGAATCGCAT** **TTGAAACTGA**

**PlumTT01**  **GCACGCAGGC** **GGTCAATTAA** **GTTAGATGTG** **AAATCCCCGG** **GCTCAACCTG** **GGAATGGCAT** **CTAAGACTGG**

**PleiATCC25** **GCATGCAGGC** **GGTCTGTTAA** **GCAAGATGTG** **AAAGCCCGGG** **GCTCAACCTC** **GGAACAGCAT** **TTTGAACTGG**

**PangATCC25** **GCATGCAGGT** **GGTCTGTTAA** **GCAAGATGTG** **AAAGCCCGGG** **GCTCAACCTC** **GGAACNGCAT** **TTTGAACTGG**

**PphoATCC11** **GCATGCAGGC** **GGTCTGTTAA** **GCAAGATGTG** **AAAGCCCGGG** **GCTCAACCTC** **GGAACAGCAT** **TTTGAACTGG**

**AfisES114**  **GCATGCAGGT** **GGTTCATTAA** **GTCAGATGTG** **AAAGCCCGGG** **GCTCAACCTC** **GGAACCGCAT** **TTGAAACTGG**

**AfisATCC77** **GCATGCAGGT** **GGTTCATTAA** **GTCAGATGTG** **AAAGCCCGGG** **GCTCAACCTC** **GGAACCGCAT** **TTGAAACTGG**

**AfisMJ11**  **GCATGCAGGT** **GGTTCATTAA** **GTCAGATGTG** **AAAGCCCGGG** **GCTCAACCTC** **GGAACCGCAT** **TTGAAACTGG**

**AfisSR7**  **GCATGCAGGT** **GGTTCATTAA** **GTCAGATGTG** **AAAGCCCGGG** **GCTCAACCTC** **GGAACCGCAT** **TTGAAACTGG**

**AfisATCC25** **GCATGCAGGT** **GGTTCATTAA** **GTCAGATGTG** **AAAGCCCGGG** **GCTCAACCTC** **GGAACCGCAT** **TTGAAACTGG**

**AlogATCC29** **GCATGCAGGT** **GGTTCATTAA** **GTCAGATGTG** **AAAGCCCGGG** **GCTCAACCTC** **GGAACCGCAT** **TTGAAACTGG**

**AlogWHSW9**  **GCATGCAGGT** **GGTTCATTAA** **GTCAGATGTG** **AAAGCCCGGG** **GCTCAACCTC** **GGAACCGCAT** **TTGAAACTGG**

**AlogWHSW1**  **GCATGCAGGT** **GGTTCATTAA** **GTCAGATGTG** **AAAGCCCGGG** **GCTCAACCTC** **GGAACCGCAT** **TTGAAACTGG**

**Asif2_4**  **GCATGCAGGT** **GGTTCATTAA** **GTCAGATGTG** **AAAGCCCGGG** **GCTCAACCTC** **GGAACCGCAT** **TTGAAACTGG**

**Asif2_5**  **GCATGCAGGT** **GGTTCATTAA** **GTCAGATGTG** **AAAGCCCGGG** **GCTCAACCTC** **GGAACCGCAT** **TTGAAACTGG**

**Asif2_6**  **GCATGCAGGT** **GGTTCATTAA** **GTCAGATGTG** **AAAGCCCGGG** **GCTCAACCTC** **GGAACCGCAT** **TTGAAACTGG**

**AthoMdR7**  **GCATGCAGGT** **GGTTCATTAA** **GTCAGATGTG** **AAAGCCCGGG** **GCTCAACCTC** **GGAACCGCAT** **TTGAAACTGG**

**AthoSA5**  **GCATGCAGGT** **GGTTCATTAA** **GTCAGATGTG** **AAAGCCCGGG** **GCTCAACCTC** **GGAACCGCAT** **TTGAAACTGG**

**AthoSA6**  **GCATGCAGGT** **GGTTCATTAA** **GTCAGATGTG** **AAAGCCCGGG** **GCTCAACCTC** **GGAACCGCAT** **TTGAAACTGG**

**Pspsp044**  **GCATGCAGGC** **GGTCTGTTAA** **GCAAGATGTG** **AAAGCCCGGG** **GCTCAACCTC** **GGAACCGCAT** **TTTGAACTGG**

**Pphosp001**  **GGATGCAGGC** **GGTCTGTTAA** **GCAAGATGTG** **AAAGCCCGGG** **GCTCAACCTC** **GGAACAGCAT** **TTTGAACTGG**

80 90 100 110 120 130 140

....|....| ....|....| ....|....| ....|....| ....|....| ....|....| ....|....|

**AsalATCC43** **TGAACTAGAG** **TGCTGTAGAG** **GGGGGTAGAA** **TTTCAGGTGT** **AGCGGTGAAA** **TGCGTAGAGA** **TCTGAAGGAA**

**AsalLFl123** **TGAACTAGAG** **TGCTGTAGAG** **GGGGGTAGAA** **TTTCAGGTGT** **AGCGGTGAAA** **TGCGTAGAGA** **TCTGAAGGAA**

**AspR863**  **TGAACTAGAG** **TGCTGTAGAG** **GGGGGTAGAA** **TTTCAGGTGT** **AGCGGTGAAA** **TGCGTAGAGA** **TCTGAAGGAA**

**AspR867**  **TGAACTAGAG** **TGCTGTAGAG** **GGGGGTAGAA** **TTTCAGGTGT** **AGCGGTGAAA** **TGCGTAGAGA** **TCTGAAGGAA**

**VspB925**  **CAAACTAGAG** **TACTGTAGAG** **GGGGGTAGAA** **TTTCAGGTGT** **AGCGGTGAAA** **TGCGTAGAGA** **TCTGAAGGAA**

**AspB915**  **TGAACTAGAG** **TGCTGTAGAG** **GGGGGTAGAA** **TTTCAGGTGT** **AGCGGTGAAA** **TGCGTAGAGA** **TCTGAAGGAA**

**AwSA12**  **TGAACTAGAG** **TGCTGTAGAG** **GGGGGTAGAA** **TTTCAGGTGT** **AGCGGTGAAA** **TGCGTAGAGA** **TCTGAAGGAA**

**AspMR1766**  **TGAACTAGAG** **TGCTGTAGAG** **GGGGGTAGAA** **TTTCAGGTGT** **AGCGGTGAAA** **TGCGTAGAGA** **TCTGAAGGAA**

**AwATCC1538** **TGAACTAGAG** **TGCTGTAGAG** **GGGGGTAGAA** **TTTCAGGTGT** **AGCGGTGAAA** **TGCGTAGAGA** **TCTGAAGGAA**

**Aw02569**  **CAAACTAGAG** **TGCTGTAGAG** **GGGGGTAGAA** **TTTCAGGTGT** **AGCGGTGAAA** **TGCGTAGAGA** **TCTGAAGGAA**

**Aw01401**  **NNAACTAGAG** **TGCTGTAGAG** **GGGGGTAGAA** **TTTCAGGTGT** **AGCGGTGAAA** **TGCGTAGAGA** **TCTGAAGGAA**

**Aw06139A**  **CAAACTAGAG** **TGCTGTAGAG** **GGGGGTAGAA** **TTTCAGGTGT** **AGCGGTGAAA** **TGCGTAGAGA** **TCTGAAGGAA**

**Al901667**  **TGAACTAGAG** **TGCTGTAGAG** **GGGGGTAGAA** **TTTCAGGTGT** **AGCGGTGAAA** **TGCGTAGAGA** **TCTGAAGGAA**

**Aw03160**  **CAAACTAGAG** **TGCTGTAGAG** **GGGGGTAGAA** **TTTCAGGTGT** **AGCGGTGAAA** **TGCGTAGAGA** **TCTGAAGGAA**

**AwSR6**  **TGAACTAGAG** **TGCTGTAGAG** **GGGGGTAGAA** **TTTCAGGTGT** **AGCGGTGAAA** **TGCGTAGAGA** **TCTGAAGGAA**

**Vt99196**  **CGGGCTAGAG** **TACTGTAGAG** **GGGGGTAGAA** **TTTCAGGTGT** **AGCGGTGAAA** **TGCGTAGAGA** **TCTGAAGGAA**

**VsplLMG190** **TGAACTAGAG** **TGCTGTAGAG** **GGGGGTAGAA** **TTTCAGGTGT** **AGCGGTGAAA** **TGCGTAGAGA** **TCTGAAGGAA**

**Vspl02066**  **TGAACTAGAG** **TGCTGTAGAG** **GGGGGTAGAA** **TTTCAGGTGT** **AGCGGTGAAA** **TGCGTAGAGA** **TCTGAAGGAA**

**Vspl04276**  **TGAACTAGAG** **TGCTGTAGAG** **GGGGGTAGAA** **TTTCAGGTGT** **AGCGGTGAAA** **TGCGTAGAGA** **TCTGAAGGAA**

**Vspl021491** **TGAACTAGAG** **TGCTGTAGAG** **GGGGGTAGAA** **TTTCAGGTGT** **AGCGGTGAAA** **TGCGTAGAGA** **TCTGAAGGAA**

**VangNB10**  **TTCACTAGAG** **TACTGTAGAG** **GGGGGTAGAA** **TTTCAGGTGT** **AGCGGTGAAA** **TGCGTAGAGA** **TCTGAAGGAA**

**GholLMG177** **CAGGCTAGAG** **TCTTGTAGAG** **GGGGGTAGAA** **TTTCAGGTGT** **AGCGGTGAAA** **TGCGTAGAGA** **TCTGAAGGAA**

**VchoATCC14** **CAAGCTAGAG** **TACTGTAGAG** **GGGGGTAGAA** **TTTCAGGTGT** **AGCGGTGAAA** **TGCGTAGAGA** **TCTGAAGGAA**

**VchoO1clas** **CAAGCTAGAG** **TACTGTAGAG** **GGGAGTAGAA** **TTTCAGGTGT** **AGCGGTGAAA** **TGCGTAGAGA** **TCTGAAGGAA**

**VchoO1E1To** **CAAGCTAGAG** **TACTGTAGAG** **GGGGGTAGAA** **TTTCAGGTGT** **AGCGGTGAAA** **TGCGTAGAGA** **TCTGAAGGAA**

**PlumTT01**  **TTGACTGGAG** **TCTCGTAGAG** **GGGGGTAGAA** **TTCCATGTGT** **AGCGGTGAAA** **TGCGTAGAGA** **TGTGGAGGAA**

**PleiATCC25** **CAGACTAGAG** **TCTTGTAGAG** **GGGGGTAGAA** **TTTCAGGTGT** **AGCGGTGAAA** **TGCGTAGAGA** **TCTGAAGGAA**

**PangATCC25** **CAGACTAGAG** **TACTGTAGAG** **GGGGGTAGAA** **TTTCAGGTGT** **AGCGGTGAAA** **TGCGTAGAGA** **TCTGAAGGAA**

**PphoATCC11** **CAGACTAGAG** **TCTTGTAGAG** **GGGGGTAGAA** **TTTCAGGTGT** **AGCGGTGAAA** **TGCGTAGAGA** **TCTGAAGGAA**

**AfisES114**  **TGAACTAGAG** **TGCTGTAGAG** **GGGGGTAGAA** **TTTCAGGTGT** **AGCGGTGAAA** **TGCGTAGAGA** **TCTGAAGGAA**

**AfisATCC77** **TGAACTAGAG** **TGCTGTAGAG** **GGGGGTAGAA** **TTTCAGGTGT** **AGCGGTGAAA** **TGCGTAGAGA** **TCTGAAGGAA**

**AfisMJ11**  **TGAACTAGAG** **TGCTGTAGAG** **GGGGGTAGAA** **TTTCAGGTGT** **AGCGGTGAAA** **TGCGTAGAGA** **TCTGAAGGAA**

**AfisSR7**  **TGAACTAGAG** **TGCTGTAGAG** **GGGGGTAGAA** **TTTCAGGTGT** **AGCGGTGAAA** **TGCGTAGAGA** **TCTGAAGGAA**

**AfisATCC25** **TGAACTAGAG** **TGCTGTAGAG** **GGGGGTAGAA** **TTTCAGGTGT** **AGCGGTGAAA** **TGCGTAGAGA** **TCTGAAGGAA**

**AlogATCC29** **TGAACTAGAG** **TGCTGTAGAG** **GGGGGTAGAA** **TTTCAGGTGT** **AGCGGTGAAA** **TGCGTAGAGA** **TCTGAAGGAA**

**AlogWHSW9**  **TGAACTAGAG** **TGCTGTAGAG** **GGGGGTAGAA** **TTTCAGGTGT** **AGCGGTGAAA** **TGCGTAGAGA** **TCTGAAGGAA**

**AlogWHSW1**  **TGAACTAGAG** **TGCTGTAGAG** **GGGGGTAGAA** **TTTCAGGTGT** **AGCGGTGAAA** **TGCGTAGAGA** **TCTGAAGGAA**

**Asif2_4**  **TGAACTAGAG** **TGCTGTAGAG** **GGGGGTAGAA** **TTTCAGGTGT** **AGCGGTGAAA** **TGCGTAGAGA** **TCTGAAGGAA**

**Asif2_5**  **TGAACTAGAG** **TGCTGTAGAG** **GGGGGTAGAA** **TTTCAGGTGT** **AGCGGTGAAA** **TGCGTAGAGA** **TCTGAAGGAA**

**Asif2_6**  **TGAACTAGAG** **TGCTGTAGAG** **GGGGGTAGAA** **TTTCAGGTGT** **AGCGGTGAAA** **TGCGTAGAGA** **TCTGAAGGAA**

**AthoMdR7**  **TGAACTAGAG** **TGCTGTAGAG** **GGGGGTAGAA** **TTTCAGGTGT** **AGCGGTGAAA** **TGCGTAGAGA** **TCTGAAGGAA**

**AthoSA5**  **TGAACTAGAG** **TGCTGTAGAG** **GGGGGTAGAA** **TTTCAGGTGT** **AGCGGTGAAA** **TGCGTAGAGA** **TCTGAAGGAA**

**AthoSA6**  **TGAACTAGAG** **TGCTGTAGAG** **GGGGGTAGAA** **TTTCAGGTGT** **AGCGGTGAAA** **TGCGTAGAGA** **TCTGAAGGAA**

**Pspsp044**  **CAGACTAGAG** **TCTTGTAGAG** **GGGGGTAGAA** **TTTCAGGTGT** **AGCGGTGAAA** **TGCGTAGAGA** **TCTGAAGGAA**

**Pphosp001**  **CAGACTAGAG** **TCTTGTAGAG** **GGGGGTAGAA** **TTTCAGGTGT** **AGCGGTGAAA** **TGCGTAGAGA** **TCTGAAGGAA**

150 160 170 180 190 200 210

....|....| ....|....| ....|....| ....|....| ....|....| ....|....| ....|....|

**AsalATCC43** **TACCAGTGGC** **GAAGGCGGCC** **CCCTGGACAG** **ACACTGACAC** **TCAGATGCGA** **AAGCGTGGGG** **AGCAAACAGG**

**AsalLFl123** **TACCAGTGGC** **GAAGGCGGCC** **CCCTGGACAG** **ACACTGACAC** **TCAGATGCGA** **AAGCGTGGGG** **AGCAAACAGG**

**AspR863**  **TACCAGTGGC** **GAAGGCGGCC** **CCCTGGACAG** **ACACTGACAC** **TCAGATGCGA** **AAGCGTGGGG** **AGCAAACAGG**

**AspR867**  **TACCAGTGGC** **GAAGGCGGCC** **CCCTGGACAG** **ACACTGACAC** **TCAGATGCGA** **AAGCGTGGGG** **AGCAAACAGG**

**VspB925**  **TACCAGTGGC** **GAAGGCGGCC** **CCCTGGACAG** **ATACTGACAC** **TCAGATGCGA** **AAGCGTGGGG** **AGCAAACAGG**

**AspB915**  **TACCAGTGGC** **GAAGGCGGCC** **CCCTGGACAG** **ACACTGACAC** **TCAGATGCGA** **AAGCGTGGGG** **AGCAAACAGG**

**AwSA12**  **TACCAGTGGC** **GAAGGCGGCC** **CCCTGGACAG** **ACACTGACAC** **TCAGATGCGA** **AAGCGTGGGG** **AGCAAACAGG**

**AspMR1766**  **TACCAGTGGC** **GAAGGCGGCC** **CCCTGGACAG** **ACACTGACAC** **TCAGATGCGA** **AAGCGTGGGG** **AGCAAACAGG**

**AwATCC1538** **TACCAGTGGC** **GAAGGCGGCC** **CCCTGGACAG** **ACACTGACAC** **TCAGATGCGA** **AAGCGTGGGG** **AGCAAACAGG**

**Aw02569**  **TACCAGTGGC** **GAAGGCGGCC** **CCCTGGACAG** **ACACTGACAC** **TCAGATGCGA** **AAGCGTGGGG** **AGCAAACAGG**

**Aw01401**  **TACCAGTGGC** **GAAGGCGGCC** **CCCTGGACAG** **ACACTGACAC** **TCAGATGCGA** **AAGCGTGGGG** **AGCAAACAGG**

**Aw06139A**  **TACCAGTGGC** **GAAGGCGGCC** **CCCTGGACAG** **ACACTGACAC** **TCAGATGCGA** **AAGCGTGGGG** **AGCAAACAGG**

**Al901667**  **TACCAGTGGC** **GAAGGCGGCC** **CCCTGGACAG** **ACACTGACAC** **TCAGATGCGA** **AAGCGTGGGG** **AGCAAACAGG**

**Aw03160**  **TACCAGTGGC** **GAAGGCGGCC** **CCCTGGACAG** **ACACTGACAC** **TCAGATGCGA** **AAGCGTGGGG** **AGCAAACAGG**

**AwSR6**  **TACCAGTGGC** **GAAGGCGGCC** **CCCTGGACAG** **ACACTGACAC** **TCAGATGCGA** **AAGCGTGGGG** **AGCAAACAGG**

**Vt99196**  **TACCAGTGGC** **GAAGGCGGCC** **CCCTGGACAG** **ATACTGACAC** **TCAGATGCGA** **AAGCGTGGGG** **AGCAAACAGG**

**VsplLMG190** **TACCAGTGGC** **GAAGGCGGCC** **CCCTGGACAG** **ACACTGACAC** **TCAGATGCGA** **AAGCGTGGGG** **AGCAAACAGG**

**Vspl02066**  **TACCAGTGGC** **GAAGGCGGCC** **CCCTGGACAG** **ACACTGACAC** **TCAGATGCGA** **AAGCGTGGGG** **AGCAAACAGG**

**Vspl04276**  **TACCAGTGGC** **GAAGGCGGCC** **CCCTGGACAG** **ACACTGACAC** **TCAGATGCGA** **AAGCGTGGGG** **AGCAAACAGG**

**Vspl021491** **TACCAGTGGC** **GAAGGCGGCC** **CCCTGGACAG** **ACACTGACAC** **TCAGATGCGA** **AAGCGTGGGG** **AGCAAACAGG**

**VangNB10**  **TACCGGTGGC** **GAAGGCGGCC** **CCCTGGACAG** **ATACTGACAC** **TCAGATGCGA** **AAGCGTGGGG** **AGCAAACAGG**

**GholLMG177** **TACCGGTGGC** **GAAGGCGGCC** **CCCTGGACAA** **AGACTGACGC** **TCAGATGCGA** **AAGCGTGGGG** **AGCAAACAGG**

**VchoATCC14** **TACCGGTGGC** **GAAGGCGGCC** **CCCTGGACAG** **ATACTGACAC** **TCAGATGCGA** **AAGCGTGGGG** **AGCAAACAGG**

**VchoO1clas** **TACCGGTGGC** **GAAGGCGGCC** **CCCTGGACAG** **ATACTGACAC** **TCAGATGCGA** **AAGCGTGGGG** **AGCAAACAGG**

**VchoO1E1To** **TACCGGTGGC** **GAAGGCGGCC** **CCCTGGACAG** **ATACTGACAC** **TCAGATGCGA** **AAGCGTGGGG** **AGCAAACAGG**

**PlumTT01**  **TACCGGTGGC** **GAAGGCGGCC** **CCCTGGACGA** **AGACTGACGC** **TCAGGTGCGA** **AAGCGTGGGG** **AGCAAACAGG**

**PleiATCC25** **TACCGGTGGC** **GAAGGCGGCC** **CCCTGGACAA** **AGACTGACGC** **TCAGATGCGA** **AAGCGTGGGG** **AGCAAACAGG**

**PangATCC25** **TACCGGTGGC** **GAAGGCGGCC** **CCCTGGACAG** **ATACTGACAC** **TCAGATGCGA** **AAGCGTGGGG** **AGCAAACAGG**

**PphoATCC11** **TACCGGTGGC** **GAAGGCGGCC** **CCCTGGACAA** **AGACTGACGC** **TCAGATGCGA** **AAGCGTGGGG** **AGCAAACAGG**

**AfisES114**  **TACCAGTGGC** **GAAGGCGGCC** **CCCTGGACAG** **ACACTGACAC** **TCAGATGCGA** **AAGCGTGGGG** **AGCAAACAGG**

**AfisATCC77** **TACCAGTGGC** **GAAGGCGGCC** **CCCTGGACAG** **ACACTGACAC** **TCAGATGCGA** **AAGCGTGGGG** **AGCAAACAGG**

**AfisMJ11**  **TACCAGTGGC** **GAAGGCGGCC** **CCCTGGACAG** **ACACTGACAC** **TCAGATGCGA** **AAGCGTGGGG** **AGCAAACAGG**

**AfisSR7**  **TACCAGTGGC** **GAAGGCGGCC** **CCCTGGACAG** **ACACTGACAC** **TCAGATGCGA** **AAGCGTGGGG** **AGCAAACAGG**

**AfisATCC25** **TACCAGTGGC** **GAAGGCGGCC** **CCCTGGACAG** **ACACTGACAC** **TCAGATGCGA** **AAGCGTGGGG** **AGCAAACAGG**

**AlogATCC29** **TACCAGTGGC** **GAAGGCGGCC** **CCCTGGACAG** **ACACTGACAC** **TCAGATGCGA** **AAGCGTGGGG** **AGCAAACAGG**

**AlogWHSW9**  **TACCAGTGGC** **GAAGGCGGCC** **CCCTGGACAG** **ACACTGACAC** **TCAGATGCGA** **AAGCGTGGGG** **AGCAAACAGG**

**AlogWHSW1**  **TACCAGTGGC** **GAAGGCGGCC** **CCCTGGACAG** **ACACTGACAC** **TCAGATGCGA** **AAGCGTGGGG** **AGCAAACAGG**

**Asif2_4**  **TACCAGTGGC** **GAAGGCGGCC** **CCCTGGACAG** **ACACTGACAC** **TCAGATGCGA** **AAGCGTGGGG** **AGCAAACAGG**

**Asif2_5**  **TACCAGTGGC** **GAAGGCGGCC** **CCCTGGACAG** **ACACTGACAC** **TCAGATGCGA** **AAGCGTGGGG** **AGCAAACAGG**

**Asif2_6**  **TACCAGTGGC** **GAAGGCGGCC** **CCCTGGACAG** **ACACTGACAC** **TCAGATGCGA** **AAGCGTGGGG** **AGCAAACAGG**

**AthoMdR7**  **TACCAGTGGC** **GAAGGCGGCC** **CCCTGGACAG** **ACACTGACAC** **TCAGATGCGA** **AAGCGTGGGG** **AGCAAACAGG**

**AthoSA5**  **TACCAGTGGC** **GAAGGCGGCC** **CCCTGGACAG** **ACACTGACAC** **TCAGATGCGA** **AAGCGTGGGG** **AGCAAACAGG**

**AthoSA6**  **TACCAGTGGC** **GAAGGCGGCC** **CCCTGGACAG** **ACACTGACAC** **TCAGATGCGA** **AAGCGTGGGG** **AGCAAACAGG**

**Pspsp044**  **TACCGGTGGC** **GAAGGCGGCC** **CCCTGGACAA** **AGACTGACGC** **TCAGATGCGA** **AAGCGTGGGG** **?G?AAACAGG**

**Pphosp001**  **TACCGGTGGC** **GAAGGCGGCC** **CCCTGGACAA** **AGACTGACGC** **TCAGATGCGA** **AAGCGTGGGG** **AGCAAACAGG**

220 230 240 250 260 270 280

....|....| ....|....| ....|....| ....|....| ....|....| ....|....| ....|....|

**AsalATCC43** **ATTAGATACC** **CTGGTAGTCC** **ACGCCGTAAA** **CGATGTCTAC** **TTGGAGGTTG** **TGGCCTTGAG** **CCGTGGCTTT**

**AsalLFl123** **ATTAGATACC** **CTGGTAGTCC** **ACGCCGTAAA** **CGATGTCTAC** **TTGGAGGTTG** **TGGCCTTGAG** **CCGTGGCTTT**

**AspR863**  **ATTAGATACC** **CTGGTAGTCC** **ACGCCGTAAA** **CGATGTCTAC** **TTGGAGGTTG** **TGGCCTTGAG** **CCGTGGCTTT**

**AspR867**  **ATTAGATACC** **CTGGTAGTCC** **ACGCCGTAAA** **CGATGTCTAC** **TTGGAGGTTG** **TGGCCTTGAG** **CCGTGGCTTT**

**VspB925**  **ATTAGATACC** **CTGGTAGTCC** **ACGCCGTAAA** **CGATGTCTAC** **TTGAAGGTTG** **TGGCCTTGAG** **CCGTGGCTTT**

**AspB915**  **ATTAGATACC** **CTGGTAGTCC** **ACGCCGTAAA** **CGATGTCTAC** **TTGGAGGTTG** **TGGCCTTGAG** **CCGTGGCTTT**

**AwSA12**  **ATTAGATACC** **CTGGTAGTCC** **ACGCCGTAAA** **CGATGTCTAC** **TTGGAGGTTG** **TGGCCTTGAG** **CCGTGGCTTT**

**AspMR1766**  **ATTAGATACC** **CTGGTAGTCC** **ACGCCGTAAA** **CGATGTCTAC** **TTGGAGGTTG** **TGGCCTTGAG** **CCGTGGCTTT**

**AwATCC1538** **ATTAGATACC** **CTGGTAGTCC** **ACGCCGTAAA** **CGATGTCTAC** **TTGGAGGTTG** **TGGCCTTGAG** **CCGTGGCTTT**

**Aw02569**  **ATTAGATACC** **CTGGTAGTCC** **ACGCCGTAAA** **CGATGTCTAC** **TTGGAGGTTG** **TGGCCTTGAG** **CCGTGGCTTT**

**Aw01401**  **ATTAGATACC** **CTGGTAGTCC** **ACGCCGTAAA** **CGATGTCTAC** **TTGGAGGTTG** **TGGCCTTGAG** **CCGTGGCTTT**

**Aw06139A**  **ATTAGATACC** **CTGGTAGTCC** **ACGCCGTAAA** **CGATGTCTAC** **TTGGAGGTTG** **TGGCCTTGAG** **CCGTGGCTTT**

**Al901667**  **ATTAGATACC** **CTGGTAGTCC** **ACGCCGTAAA** **CGATGTCTAC** **TTGGAGGTTG** **TGGCCTTGAG** **CCGTGGCTTT**

**Aw03160**  **ATTAGATACC** **CTGGTAGTCC** **ACGCCGTAAA** **CGATGTCTAC** **TTGGAGGTTG** **TGGCCTTGAG** **CCGTGGCTTT**

**AwSR6**  **ATTAGATACC** **CTGGTAGTCC** **ACGCCGTAAA** **CGATGTCTAC** **TTGGAGGTTG** **TGGCCTTGAG** **CCGTGGCTTT**

**Vt99196**  **ATTAGATACC** **CTGGTAGTCC** **ACGCCGTAAA** **CGATGTCTAC** **TTGGAGGTTG** **TGGCCTTGAG** **CCGTGGCTTT**

**VsplLMG190** **ATTAGATACC** **CTGGTAGTCC** **ACGCCGTAAA** **CGATGTCTAC** **TTGGAGGTTG** **TGGCCTTGAG** **CCGTGGCTTT**

**Vspl02066**  **ATTAGATACC** **CTGGTAGTCC** **ACGCCGTAAA** **CGATGTCTAC** **TTGGAGGTTG** **TGGCCTTGAG** **CCGTGGCTTT**

**Vspl04276**  **ATTAGATACC** **CTGGTAGTCC** **ACGCCGTAAA** **CGATGTCTAC** **TTGGAGGTTG** **TGGCCTTGAG** **CCGTGGCTTT**

**Vspl021491** **ATTAGATACC** **CTGGTAGTCC** **ACGCCGTAAA** **CGATGTCTAC** **TTGGAGGTTG** **TGGCCTTGAG** **CCGTGGCTTT**

**VangNB10**  **ATTAGATACC** **CTGGTAGTCC** **ACGCCGTAAA** **CGATGTCTAC** **TTGGAGGTTG** **TGGCCTTGAG** **CCGTGGCTTT**

**GholLMG177** **ATTAGATACC** **CTGGTAGTCC** **ACGCTGTAAA** **CGATGTCTAC** **TTGGAGGCTG** **TGGTCTAGAA** **CCGTGGCTTT**

**VchoATCC14** **ATTAGATACC** **CTGGTAGTCC** **ACGCCGTAAA** **CGATGTCTAC** **TTGGAGGTTG** **TG?CCTAGAG** **??GTGGCTTT**

**VchoO1clas** **ATTAGATACC** **CTGGTAGTCC** **ACGCCGTAAA** **CGATGTCTAC** **TTGGAGGTTG** **TGCCCTAGAG** **GTGTGGCTTT**

**VchoO1E1To** **ATTAGATACC** **CTGGTAGTCC** **ACGCCGTAAA** **CGATGTCTAC** **TTGGAGGTTG** **TGCCCTAGAG** **GTGTGGCTTT**

**PlumTT01**  **ATTAGATACC** **CTGGTAGTCC** **ACGCTGTAAA** **CGATGTCGAT** **TTGGAGGTTG** **CGGTCTTGAA** **CGGTGGCTTC**

**PleiATCC25** **ATTAGATACC** **CTGGTAGTCC** **ACGCCGTAAA** **CGATGTCTAC** **TTGGAGGTTG** **TGGCCTTGAG** **CCGTGGCTTT**

**PangATCC25** **ATTAGATACC** **CTGGTAGTCC** **ACGCCGTAAA** **CGATGTCTAC** **TTGGAGGTTG** **TGGCCTTGAG** **CCGTGGCTTT**

**PphoATCC11** **ATTAGATACC** **CTGGTAGTCC** **ACGCCGTAAA** **CGATGTCTAC** **TTGAAGGTTG** **TGGCCTTGAG** **CCGTGGCTTT**

**AfisES114**  **ATTAGATACC** **CTGGTAGTCC** **ACGCCGTAAA** **CGATGTCTAC** **TTGGAGGTTG** **TTCCCTTGAG** **GAGTGGCTTT**

**AfisATCC77** **ATTAGATACC** **CTGGTAGTCC** **ACGCCGTAAA** **CGATGTCTAC** **TTGGAGGTTG** **TTCCCTTGAG** **GAGTGGCTTT**

**AfisMJ11**  **ATTAGATACC** **CTGGTAGTCC** **ACGCCGTAAA** **CGATGTCTAC** **TTGGAGGTTG** **TTCCCTTGAG** **GAGTGGCTTT**

**AfisSR7**  **ATTAGATACC** **CTGGTAGTCC** **ACGCCGTAAA** **CGATGTCTAC** **TTGGAGGTTG** **TTCCCTTGAG** **GAGTGGCTTT**

**AfisATCC25** **ATTAGATACC** **CTGGTAGTCC** **ACGCCGTAAA** **CGATGTCTAC** **TTGGAGGTTG** **TTCCCTTGAG** **GAGTGGCTTT**

**AlogATCC29** **ATTAGATACC** **CTGGTAGTCC** **ACGCCGTAAA** **CGATGTCTAC** **TTGGAGGTTG** **TGGCCTTGAG** **CCGTGGCTTT**

**AlogWHSW9**  **ATTAGATACC** **CTGGTAGTCC** **ACGCCGTAAA** **CGATGTCTAC** **TTGGAGGTTG** **TGGCCTTGAG** **CCGTGGCTTT**

**AlogWHSW1**  **ATTAGATACC** **CTGGTAGTCC** **ACGCCGTAAA** **CGATGTCTAC** **TTGGAGGTTG** **TGGCCTTGAG** **CCGTGGCTTT**

**Asif2_4**  **ATTAGATACC** **CTGGTAGTCC** **ACGCCGTAAA** **CGATGTCTAC** **TTGGAGGTTG** **TGGCCTTGAG** **CCGTGGCTTT**

**Asif2_5**  **ATTAGATACC** **CTGGTAGTCC** **ACGCCGTAAA** **CGATGTCTAC** **TTGGAGGTTG** **TGGCCTTGAG** **CCGTGGCTTT**

**Asif2_6**  **ATTAGATACC** **CTGGTAGTCC** **ACGCCGTAAA** **CGATGTCTAC** **TTGGAGGTTG** **TGGCCTTGAG** **CCGTGGCTTT**

**AthoMdR7**  **ATTAGATACC** **CTGGTAGTCC** **ACGCCGTAAA** **CGATGTCTAC** **TTGGAGGTTG** **TGGCCTTGAG** **CCGTGGCTTT**

**AthoSA5**  **ATTAGATACC** **CTGGTAGTCC** **ACGCCGTAAA** **CGATGTCTAC** **TTGGAGGTTG** **TGGCCTTGAG** **CCGTGGCTTT**

**AthoSA6**  **ATTAGATACC** **CTGGTAGTCC** **ACGCCGTAAA** **CGATGTCTAC** **TTGGAGGTTG** **TGGCCTTGAG** **CCGTGGCTTT**

**Pspsp044**  **ATTAGATACC** **CTGGTAGTCC** **ACGCCGTAAA** **CGATGTCTAC** **TTGAAGGTTG** **TGGCCTTGAG** **CCGTGGCTTT**

**Pphosp001**  **ATTAGATACC** **CTGGTAGTCC** **ACGCCGTAAA** **CGATGTCTAC** **TTGAAGGTTG** **TGGCCTTGAG** **CCGTGGCTTT**

290 300 310 320 330 340 350

....|....| ....|....| ....|....| ....|....| ....|....| ....|....| ....|....|

**AsalATCC43** **CGGAGCTAAC** **GCGTTAAGTA** **GACCGCCTGG** **GGAGTACGGT** **CGCAAGATTA** **AAACTCAAAT** **GAATTGACGG**

**AsalLFl123** **CGGAGCTAAC** **GCGTTAAGTA** **GACCGCCTGG** **GGAGTACGGT** **CGCAAGATTA** **AAACTCAAAT** **GAATTGACGG**

**AspR863**  **CGGAGCTAAC** **GCGTTAAGTA** **GACCGCCTGG** **GGAGTACGGT** **CGCAAGATTA** **AAACTCAAAT** **GAATTGACGG**

**AspR867**  **CGGAGCTAAC** **GCGTTAAGTA** **GACCGCCTGG** **GGAGTACGGT** **CGCAAGATTA** **AAACTCAAAT** **GAATTGACGG**

**VspB925**  **CGGAGCTAAC** **GCGTTAAGTA** **GACCGCCTGG** **GGAGTACGGT** **CGCAAGATTA** **AAACTCAAAT** **GAATTGACGG**

**AspB915**  **CGGAGCTAAC** **GCGTTAAGTA** **GACCGCCTGG** **GGAGTACGGT** **CGCAAGATTA** **AAACTCAAAT** **GAATTGACGG**

**AwSA12**  **CGGAGCTAAC** **GCGTTAAGTA** **GACCGCCTGG** **GGAGTACGGT** **CGCAAGATTA** **AAACTCAAAT** **GAATTGACGG**

**AspMR1766**  **CGGAGCTAAC** **GCGTTAAGTA** **GACCGCCTGG** **GGAGTACGGT** **CGCAAGATTA** **AAACTCAAAT** **GAATTGACGG**

**AwATCC1538** **CGGAGCTAAC** **GCGTTAAGTA** **GACCGCCTGG** **GGAGTACGGT** **CGCAAGATTA** **AAACTCAAAT** **GAATTGACGG**

**Aw02569**  **CGGAGCTAAC** **GCGTTAAGTA** **GACCGCCTGG** **GGAGTACGGT** **CGCAAGATTA** **AAACTCAAAT** **GAATTGACGG**

**Aw01401**  **CGGAGCTAAC** **GCGTTAAGTA** **GACCGCCTGG** **GGAGTACGGT** **CGCAAGATTA** **AAACTCAAAT** **GAATTGACGG**

**Aw06139A**  **CGGAGCTAAC** **GCGTTAAGTA** **GACCGCCTGG** **GGAGTACGGT** **CGCAAGATTA** **AAACTCAAAT** **GAATTGACGG**

**Al901667**  **CGGAGCTAAC** **GCGTTAAGTA** **GACCGCCTGG** **GGAGTACGGT** **CGCAAGATTA** **AAACTCAAAT** **GAATTGACGG**

**Aw03160**  **CGGAGCTAAC** **GCGTTAAGTA** **GACCGCCTGG** **GGAGTACGGT** **CGCAAGATTA** **AAACTCAAAT** **GAATTGACGG**

**AwSR6**  **CGGAGCTAAC** **GCGTTAAGTA** **GACCGCCTGG** **GGAGTACGGT** **CGCAAGATTA** **AAACTCAAAT** **GAATTGACGG**

**Vt99196**  **CGGAGCTAAC** **GCGTTAAGTA** **GACCGCCTGG** **GGAGTACGGT** **CGCAAGATTA** **AAACTCAAAT** **GAATTGACGG**

**VsplLMG190** **CGGAGCTAAC** **GCGTTAAGTA** **GACCGCCTGG** **GGAGTACGGT** **CGCAAGATTA** **AAACTCAAAT** **GAATTGACGG**

**Vspl02066**  **CGGAGCTAAC** **GCGTTAAGTA** **GACCGCCTGG** **GGAGTACGGT** **CGCAAGATTA** **AAACTCAAAT** **GAATTGACGG**

**Vspl04276**  **CGGAGCTAAC** **GCGTTAAGTA** **GACCGCCTGG** **GGAGTACGGT** **CGCAAGATTA** **AAACTCAAAT** **GAATTGACGG**

**Vspl021491** **CGGAGCTAAC** **GCGTTAAGTA** **GACCGCCTGG** **GGAGTACGGT** **CGCAAGATTA** **AAACTCAAAT** **GAATTGACGG**

**VangNB10**  **CGGAGCTAAC** **GCGTTAAGTA** **GACCGCCTGG** **GGAGTACGGT** **CGCAAGATTA** **AAACTCAAAT** **GAATTGACGG**

**GholLMG177** **CGGAGCTAAC** **GCGTTAAGTA** **GACCGCCTGG** **GGAGTACGGT** **CGCAAGATTA** **AAACTCAAAT** **GAATTGACGG**

**VchoATCC14** **CGGAGCTAAC** **GCGTTAAGTA** **GACCGCCTGG** **GGAGTACGGT** **CGCAAGATTA** **AAACTCAAAT** **GAATTGACGG**

**VchoO1clas** **CGGAGCTAAC** **GCGTTAAGTA** **GACCGCCTGG** **GGAGTACGGT** **CGCAAGATTA** **AAACTCAAAT** **GAATTGACGG**

**VchoO1E1To** **CGGAGCTAAC** **GCGTTAAGTA** **GACCGCCTGG** **GGAGTACGGT** **CGCAAGATTA** **AAACTCAAAT** **GAATTGACGG**

**PlumTT01**  **CGAAGCTAAC** **GCGTTAAATC** **GACCGCCTGG** **GGAGTACGGC** **CGCAAGGTTA** **AAACTCAAAT** **GAATTGACGG**

**PleiATCC25** **CGGAGCTAAC** **GCGTTAAGTA** **GACCGCCTGG** **GGAGTACGGT** **CGCAAGATTA** **AAACTCAAAT** **GAATTGACGG**

**PangATCC25** **CGGAGCTAAC** **GCGTTAAGTA** **GACCGCCTGG** **GGAGTACGGT** **CGCAAGATTA** **AAACTCAAAT** **GAATTGACGG**

**PphoATCC11** **CGGAGCTAAC** **GCGTTAAGTA** **GACCGCCTGG** **GGAGTACGGT** **CGCAAGATTA** **AAACTCAAAT** **GAATTGACGG**

**AfisES114**  **CGGAGCTAAC** **GCGTTAAGTA** **GACCGCCTGG** **GGAGTACGGT** **CGCAAGATTA** **AAACTCAAAT** **GAATTGACGG**

**AfisATCC77** **CGGAGCTAAC** **GCGTTAAGTA** **GACCGCCTGG** **GGAGTACGGT** **CGCAAGATTA** **AAACTCAAAT** **GAATTGACGG**

**AfisMJ11**  **CGGAGCTAAC** **GCGTTAAGTA** **GACCGCCTGG** **GGAGTACGGT** **CGCAAGATTA** **AAACTCAAAT** **GAATTGACGG**

**AfisSR7**  **CGGAGCTAAC** **GCGTTAAGTA** **GACCGCCTGG** **GGAGTACGGT** **CGCAAGATTA** **AAACTCAAAT** **GAATTGACGG**

**AfisATCC25** **CGGAGCTAAC** **GCGTTAAGTA** **GACCGCCTGG** **GGAGTACGGT** **CGCAAGATTA** **AAACTCAAAT** **GAATTGACGG**

**AlogATCC29** **CGGAGCTAAC** **GCGTTAAGTA** **GACCGCCTGG** **GGAGTACGGT** **CGCAAGATTA** **AAACTCAAAT** **GAATTGACGG**

**AlogWHSW9**  **CGGAGCTAAC** **GCGTTAAGTA** **GACCGCCTGG** **GGAGTACGGT** **CGCAAGATTA** **AAACTCAAAT** **GAATTGACGG**

**AlogWHSW1**  **CGGAGCTAAC** **GCGTTAAGTA** **GACCGCCTGG** **GGAGTACGGT** **CGCAAGATTA** **AAACTCAAAT** **GAATTGACGG**

**Asif2_4**  **CGGAGCTAAC** **GCGTTAAGTA** **GACCGCCTGG** **GGAGTACGGT** **CGCAAGATTA** **AAACTCAAAT** **GAATTGACGG**

**Asif2_5**  **CGGAGCTAAC** **GCGTTAAGTA** **GACCGCCTGG** **GGAGTACGGT** **CGCAAGATTA** **AAACTCAAAT** **GAATTGACGG**

**Asif2_6**  **CGGAGCTAAC** **GCGTTAAGTA** **GACCGCCTGG** **GGAGTACGGT** **CGCAAGATTA** **AAACTCAAAT** **GAATTGACGG**

**AthoMdR7**  **CGGAGCTAAC** **GCGTTAAGTA** **GACCGCCTGG** **GGAGTACGGT** **CGCAAGATTA** **AAACTCAAAT** **GAATTGACGG**

**AthoSA5**  **CGGAGCTAAC** **GCGTTAAGTA** **GACCGCCTGG** **GGAGTACGGT** **CGCAAGATTA** **AAACTCAAAT** **GAATTGACGG**

**AthoSA6**  **CGGAGCTAAC** **GCGTTAAGTA** **GACCGCCTGG** **GGAGTACGGT** **CGCAAGATTA** **AAACTCAAAT** **GAATTGACGG**

**Pspsp044**  **CGGAGCTAAC** **GCGTTAAGTA** **GACCGCCTGG** **GGAGTACGGT** **CGCAAGATTA** **AAACTCAAAT** **GAATTGACGG**

**Pphosp001**  **CGGAGCTAAC** **GCGTTAAGTA** **GACCGCCTGG** **GGAGTACGGT** **CGCAAGATTA** **AAACTCAAAT** **GAATTGACGG**

360 370 380 390 400 410 420

....|....| ....|....| ....|....| ....|....| ....|....| ....|....| ....|....|

**AsalATCC43** **GGGCC-GCAC** **AAGCGGTGGA** **GCATGTGGTT** **TAATTCGATG** **CAACGCGAAG** **AACCTTACCT** **ACTCTTGACA**

**AsalLFl123** **GGGCCCGCAC** **AAGCGGTGGA** **GCATGTGGTT** **TAATTCGATG** **CAACGCGAAG** **AACCTTACCT** **ACTCTTGACA**

**AspR863**  **GGGCCCGCAC** **AAGCGGTGGA** **GCATGTGGTT** **TAATTCGATG** **CAACGCGAAG** **AACCTTACCT** **ACTCTTGACA**

**AspR867**  **GGGCCCGCAC** **AAGCGGTGGA** **GCATGTGGTT** **TAATTCGATG** **CAACGCGAAG** **AACCTTACCT** **ACTCTTGACA**

**VspB925**  **GGGCCCGCAC** **AAGCGGTGGA** **GCATGTGGTT** **TAATTCGATG** **CAACGCGAAG** **AACCTTACCT** **ACTCTTGACA**

**AspB915**  **GGGCCCGCAC** **AAGCGGTGGA** **GCATGTGGTT** **TAATTCGATG** **CAACGCGAAG** **AACCTTACCT** **ACTCTTGACA**

**AwSA12**  **GGGCCCGCAC** **AAGCGGTGGA** **GCATGTGGTT** **TAATTCGATG** **CAACGCGAAG** **AACCTTACCT** **ACTCTTGACA**

**AspMR1766**  **GGGCCCGCAC** **AAGCGGTGGA** **GCATGTGGTT** **TAATTCGATG** **CAACGCGAAG** **AACCTTACCT** **ACTCTTGACA**

**AwATCC1538** **GGGCCCGCAC** **AAGCGGTGGA** **GCATGTGGTT** **TAATTCGATG** **CAACGCGAAG** **AACCTTACCT** **ACTCTTGACA**

**Aw02569**  **GGGCCCGCAC** **AAGCGGTGGA** **GCATGTGGTT** **TAATTCGATG** **CAACGCGAAG** **AACCTTACCT** **ACTCTTGACA**

**Aw01401**  **GGGCCCGCAC** **AAGCGGTGGA** **GCATGTGGTT** **TAATTCGATG** **CAACGCGAAG** **AACCTTACCT** **ACTCTTGACA**

**Aw06139A**  **GGGCCCGCAC** **AAGCGGTGGA** **GCATGTGGTT** **TAATTCGATG** **CAACGCGAAG** **AACCTTACCT** **ACTCTTGACA**

**Al901667**  **GGGCCCGCAC** **AAGCGGTGGA** **GCATGTGGTT** **TAATTCGATG** **CAACGCGAAG** **AACCTTACCT** **ACTCTTGACA**

**Aw03160**  **GGGCCCGCAC** **AAGCGGTGGA** **GCATGTGGTT** **TAATTCGATG** **CAACGCGAAG** **AACCTTACCT** **ACTCTTGACA**

**AwSR6**  **GGGCCCGCAC** **AAGCGGTGGA** **GCATGTGGTT** **TAATTCGATG** **CAACGCGAAG** **AACCTTACCT** **ACTCTTGACA**

**Vt99196**  **GGGCCCGCAC** **AAGCGGTGGA** **GCATGTGGTT** **TAATTCGATG** **CAACGCGAAG** **AACCTTACCT** **ACTCTTGACA**

**VsplLMG190** **GGGCCCGCAC** **AAGCGGTGGA** **GCATGTGGTT** **TAATTCGATG** **CAACGCGAAG** **AACCTTACCT** **ACTCTTGACA**

**Vspl02066**  **GGGCCCGCAC** **AAGCGGTGGA** **GCATGTGGTT** **TAATTCGATG** **CAACGCGAAG** **AACCTTACCT** **ACTCTTGACA**

**Vspl04276**  **GGGCCCGCAC** **AAGCGGTGGA** **GCATGTGGTT** **TAATTCGATG** **CAACGCGAAG** **AACCTTACCT** **ACTCTTGACA**

**Vspl021491** **GGGCCCGCAC** **AAGCGGTGGA** **GCATGTGGTT** **TAATTCGATG** **CAACGCGAAG** **AACCTTACCT** **ACTCTTGACA**

**VangNB10**  **GGGCCCGCAC** **AAGCGGTGGA** **GCATGTGGTT** **TAATTCGATG** **CAACGCGAAG** **AACCTTACCT** **ACTCTTGACA**

**GholLMG177** **GGGCCCGCAC** **AAGCGGTGGA** **GCATGTGGTT** **TAATTCGATG** **CAACGCGAAG** **AACCTTACCT** **ACTCTTGACA**

**VchoATCC14** **GGGCCCGCAC** **AAGCGGTGGA** **GCATGTGGTT** **TAATTCGATG** **CAACGCGAAG** **AACCTTACCT** **ACTCTTGACA**

**VchoO1clas** **GGGCCCGCAC** **AAGCGGTGGA** **GCATGTGGTT** **TAATTCGATG** **CAACGCGAAG** **AACCTTACCT** **ACTCTTGACA**

**VchoO1E1To** **GGGCCCGCAC** **AAGCGGTGGA** **GCATGTGGTT** **TAATTCGATG** **CAACGCGAAG** **AACCTTACCT** **ACTCTTGACA**

**PlumTT01**  **GGGCCCGCAC** **AAGCGGTGGA** **GCATGTGGTT** **TAATTCGATG** **CAACGCGAAG** **AACCTTACCT** **ACTCTTGACA**

**PleiATCC25** **GGGNCCGCAC** **AAGCGGTGGA** **GCATGTGGTT** **TAATTCGANG** **CAACGCGAAG** **AACCTTACCT** **ACTCTTGACA**

**PangATCC25** **GGGNCCGCAC** **AAGCGGTGGA** **GCATGTGGTT** **TAATTCGANG** **CAACGCGAAG** **AACCTTACCT** **ACTCTTGACA**

**PphoATCC11** **GGGCCCGCAC** **AAGCGGTGGA** **GCATGTGGTT** **TAATTCGATG** **CAACGCGAAG** **AACCTTACCT** **ACTCTTGACA**

**AfisES114**  **GGGCCCGCAC** **AAGCGGTGGA** **GCATGTGGTT** **TAATTCGATG** **CAACGCGAAG** **AACCTTACCT** **ACTCTTGACA**

**AfisATCC77** **GGGCCCGCAC** **AAGCGGTGGA** **GCATGTGGTT** **TAATTCGATG** **CAACGCGAAG** **AACCTTACCT** **ACTCTTGACA**

**AfisMJ11**  **GGGCCCGCAC** **AAGCGGTGGA** **GCATGTGGTT** **TAATTCGATG** **CAACGCGAAG** **AACCTTACCT** **ACTCTTGACA**

**AfisSR7**  **GGGCCCGCAC** **AAGCGGTGGA** **GCATGTGGTT** **TAATTCGATG** **CAACGCGAAG** **AACCTTACCT** **ACTCTTGACA**

**AfisATCC25** **GGGCCCGCAC** **AAGCGGTGGA** **GCATGTGGTT** **TAATTCGATG** **CAACGCGAAG** **AACCTTACCT** **ACTCTTGACA**

**AlogATCC29** **GGGCC-GCAC** **AAGCGGTGGA** **GCATGTGGTT** **TAATTCGATG** **CAACGCGAAG** **AACCTTACCT** **ACTCTTGACA**

**AlogWHSW9**  **GGGCCCGCAC** **AAGCGGTGGA** **GCATGTGGTT** **TAATTCGATG** **CAACGCGAAG** **AACCTTACCT** **ACTCTTGACA**

**AlogWHSW1**  **GGGCCCGCAC** **AAGCGGTGGA** **GCATGTGGTT** **TAATTCGATG** **CAACGCGAAG** **AACCTTACCT** **ACTCTTGACA**

**Asif2_4**  **GGGCCCGCAC** **AAGCGGTGGA** **GCATGTGGTT** **TAATTCGATG** **CAACGCGAAG** **AACCTTACCT** **ACTCTTGACA**

**Asif2_5**  **GGGCCCGCAC** **AAGCGGTGGA** **GCATGTGGTT** **TAATTCGATG** **CAACGCGAAG** **AACCTTACCT** **ACTCTTGACA**

**Asif2_6**  **GGGCCCGCAC** **AAGCGGTGGA** **GCATGTGGTT** **TAATTCGATG** **CAACGCGAAG** **AACCTTACCT** **ACTCTTGACA**

**AthoMdR7**  **GGGCCCGCAC** **AAGCGGTGGA** **GCATGTGGTT** **TAATTCGATG** **CAACGCGAAG** **AACCTTACCT** **ACTCTTGACA**

**AthoSA5**  **GGGCCCGCAC** **AAGCGGTGGA** **GCATGTGGTT** **TAATTCGATG** **CAACGCGAAG** **AACCTTACCT** **ACTCTTGACA**

**AthoSA6**  **GGGCCCGCAC** **AAGCGGTGGA** **GCATGTGGTT** **TAATTCGATG** **CAACGCGAAG** **AACCTTACCT** **ACTCTTGACA**

**Pspsp044**  **GGGCCCGCAC** **AAGCGGTGGA** **GCATGTGGTT** **TAATTCGATG** **CAACGCGAAG** **AACCTTACCT** **ACTCTTGACA**

**Pphosp001**  **GGGCCCGCAC** **AAGCGGTGGA** **GCATGTGGTT** **TAATTCGATG** **CAACGCGAAG** **AACCTTACCT** **ACTCTTGACA**

430 440 450 460 470 480 490

....|....| ....|....| ....|....| ....|....| ....|....| ....|....| ....|....|

**AsalATCC43** **TCTACAGAAT** **TCGCTAGAGA** **TAGCTTAGTG** **CCTTCGGGAA** **CTGTAAGACA** **GGTGCTGCAT** **GGCTGTCGTC**

**AsalLFl123** **TCTACAGAAT** **TCGCTAGAGA** **TAGCTTAGTG** **CCTTCGGGAA** **CTGTAAGACA** **GGTGCTGCAT** **GGCTGTCGTC**

**AspR863**  **TCCACAGAAG** **AGACCAGAGA** **TGGACTTGTG** **CCTTCGGGAA** **CTGTGAGACA** **GGTGCTGCAT** **GGCTGTCGTC**

**AspR867**  **TCCACAGAAG** **AGACCAGAGA** **TGGACTTGTG** **CCTTCGGGAA** **CTGTGAGACA** **GGTGCTGCAT** **GGCTGTCGTC**

**VspB925**  **TCCAGAGAAG** **CCAGTGGAGA** **CACAGGCGTG** **CCTTCGGGAG** **CTCTGAGACA** **GGTGCTGCAT** **GGCTGTCGTC**

**AspB915**  **TCTACAGAAT** **TCGCTAGAGA** **TAGCTTAGTG** **CCTTCGGGAA** **CTGTAAGACA** **GGTGCTGCAT** **GGCTGTCGTC**

**AwSA12**  **TCCACAGAAT** **TCGCTAGAGA** **TAGCTTAGTG** **CCTTCGGGAA** **CTGTGAGACA** **GGTGCTGCAT** **GGCTGTCGTC**

**AspMR1766**  **TCTACAGAAT** **TCGCTAGAGA** **TAGCTTAGTG** **CCTTCGGGAA** **CTGTAAGACA** **GGTGCTGCAT** **GGCTGTCGTC**

**AwATCC1538** **TCTACAGAAT** **TCGCTAGAGA** **TAGCTTAGTG** **CCTTCGGGAA** **CTGTAAGACA** **GGTGCTGCAT** **GGCTGTCGTC**

**Aw02569**  **TCCAGAGAAT** **TCGCTAGAGA** **TAGCTTAGTG** **CCTTCGGGAG** **CTCTGAGACA** **GGTGCTGCAT** **GGCTGTCGTC**

**Aw01401**  **TCCAGAGAAT** **TCGCTAGAGA** **TAGCTTAGTG** **CCTTCGGGAA** **CTCTGAGACA** **GGTGCTGCAT** **GGCTGTCGTC**

**Aw06139A**  **TCCAGAGAAT** **TCGCTAGAGA** **TAGCTTAGTG** **CCTTCGGGAG** **CTCTGAGACA** **GGTGCTGCAT** **GGCTGTCGTC**

**Al901667**  **TCTACAGAAT** **TCGCTAGAGA** **TAGCTTAGTG** **CCTTCGGGAA** **CTGTAAGACA** **GGTGCTGCAT** **GGCTGTCGTC**

**Aw03160**  **TCCAGAGAAT** **TCGCTAGAGA** **TAGCTTAGTG** **CCTTCGGGAG** **CTCTGAGACA** **GGTGCTGCAT** **GGCTGTCGTC**

**AwSR6**  **TCTACAGAAT** **TCGCTAGAGA** **TAGCTTAGTG** **CCTTCGGGAA** **CTGTAAGACA** **GGTGCTGCAT** **GGCTGTCGTC**

**Vt99196**  **TCCAGAGAAG** **CCAGCGGAGA** **CGCAGGTGTG** **CCTTCGGGAA** **CTCTGAGACA** **GGTGCTGCAT** **GGCTGTCGTC**

**VsplLMG190** **TCCAGAGAAG** **CCAGCGGAGA** **CGCAGGTGTG** **CCTTCGGGAA** **CTCTGAGACA** **GGTGCTGCAT** **GGCTGTCGTC**

**Vspl02066**  **TCCAGAGAAG** **CCAGCGGAGA** **CGCAGGTGTG** **CCTTCGGGAA** **CTCTGAGACA** **GGTGCTGCAT** **GGCTGTCGTC**

**Vspl04276**  **TCCAGAGAAG** **CCAGCGGAGA** **CGCAGGTGTG** **CCTTCGGGAA** **CTCTGAGACA** **GGTGCTGCAT** **GGCTGTCGTC**

**Vspl021491** **TCCAGAGAAT** **CCAGCGGAGA** **CGCAGGTGTG** **CCTTCGGGAA** **CTCTGAGACA** **GGTGCTGCAT** **GGCTGTCGTC**

**VangNB10**  **TCCAGAGAAG** **CCAGCGGAGA** **CGCAGGTGTG** **CCTTCGGGAG** **CTCTGAGACA** **GGTGCTGCAT** **GGCTGTCGTC**

**GholLMG177** **TCCTCAGAAG** **CCGGAAGAGA** **TTCTGGTGTG** **CCTTCGGGAA** **CTGAGAGACA** **GGTGCTGCAT** **GGCTGTCGTC**

**VchoATCC14** **TCCAGAGAAT** **CT?GCGGAGA** **CGCTGGAGTG** **CCTTCGGGAG** **CTCTGAGACA** **GGTGCTGCAT** **GGCTGTCGTC**

**VchoO1clas** **TCCAGAGAAT** **CTAGCGGAGA** **CGCTGGAGTG** **CCTTCGGGAG** **CTCTGAGACA** **GGTGCTGCAT** **GGCTGTCGTC**

**VchoO1E1To** **TCCAGAGAAT** **CTAGCGGAGA** **CGCTGGAGTG** **CCTTCGGGAG** **CTCTGAGACA** **GGTGCTGCAT** **GGCTGTCGTC**

**PlumTT01**  **TCCTCAGAAT** **TTGCTGGAGA** **CAGCGAAGTG** **CCTTAGGGAA** **CTGAGAGACA** **GGTGCTGCAT** **GGCTGTCGTC**

**PleiATCC25** **TCCAGAGAAC** **TTTCCAGAGA** **TGCATTGGTG** **CCTTCGGGAA** **CTCTGAGACA** **GGTGCTGCAT** **GGCTGTCGTC**

**PangATCC25** **TCCANAGAAC** **TTTCCAGAGA** **TAGATTGGTG** **CCTTCGGGAA** **CTCTGAGACA** **GGTGCTGCAT** **GGCTGTCGTC**

**PphoATCC11** **TCCAGAGAAT** **TCGCTAGAGA** **TAGCTTAGTG** **CCTTCGGGAA** **CTCTGAGACA** **GGTGCTGCAT** **GGCTGTCGTC**

**AfisES114**  **TCCAGAGAAT** **TCGCTAGAGA** **TAGCTTAGTG** **CCTTCGGGAA** **CTCTGAGACA** **GGTGCTGCAT** **GGCTGTCGTC**

**AfisATCC77** **TCCAGAGAAT** **TCGCTAGAGA** **TAGCTTAGTG** **CCTTCGGGA?** **CTCTGAGACA** **GGTGCTGCAT** **GGCTGTCGTC**

**AfisMJ11**  **TCCAGAGAAT** **TCGCTAGAGA** **TAGCTTAGTG** **CCTTCGGGA?** **CTCTGAGACA** **GGTGCTGCAT** **GGCTGTCGTC**

**AfisSR7**  **TCCAGAGAAT** **T?GCTAGAGA** **TAGCTTAGTG** **CCTTCGGGAG** **CTCTGAGACA** **GGTGCTGCAT** **GGCTGTCGTC**

**AfisATCC25** **TCCAGAGAAT** **TCGCTAGAGA** **TAGCTTAGTG** **CCTTCGGGAG** **CTCTGAGACA** **GGTGCTGCAT** **GGCTGTCGTC**

**AlogATCC29** **TCTACAGAAT** **TNNCTAGAGA** **TAGNTTAGTG** **CCTTCGGGAA** **CTGTAAGACA** **GGTGCTGCAT** **GGCTGTCGTC**

**AlogWHSW9**  **TCTACAGAAT** **TCGCTAGAGA** **TAGCTTAGTG** **CCTTCGGGAA** **CTGTAAGACA** **GGTGCTGCAT** **GGCTGTCGTC**

**AlogWHSW1**  **TCTACAGAAT** **TCGCTAGAGA** **TAGCTTAGTG** **CCTTCGGGAA** **CTGTAAGACA** **GGTGCTGCAT** **GGCTGTCGTC**

**Asif2_4**  **TCCA?AGAAT** **TCGCTAGAGA** **TAGCTTAGTG** **CCTTCGGGA?** **CT?TGAGACA** **GGTGCTGCAT** **GGCTGTCGTC**

**Asif2_5**  **TCCA?AGAAT** **TCGCTAGAGA** **TAGCTTAGTG** **CCTTCGGGA?** **CT?TGAGACA** **GGTGCTGCAT** **GGCTGTCGTC**

**Asif2_6**  **TCCA?AGAAT** **TCGCTAGAGA** **TAGCTTAGTG** **CCTTCGGGAA** **CT?TGAGACA** **GGTGCTGCAT** **GGCTGTCGTC**

**AthoMdR7**  **TCCAGAGAAT** **TCGCTAGAGA** **TAGCTTA?TG** **CCTTCGGGAG** **CTCTGAGACA** **GGTGCTGCAT** **GGCTGTCGTC**

**AthoSA5**  **TCCAGAGAAT** **TCGCTAGAGA** **TAGCTTAGTG** **CCTTCGGGAG** **CTCTGAGACA** **GGTGCTGCAT** **GGCTGTCGTC**

**AthoSA6**  **TCCAGAGAAT** **TCGCTAGAGA** **TAGCTTAGTG** **CCTTCGGGA?** **CTCT?AGACA** **GGTGCTGCAT** **GGCTGTCGTC**

**Pspsp044**  **TCCAGAGAAT** **TCGCTAGAGA** **TAGCTTAGTG** **CCTTCGGGAA** **CTCTGAGACA** **GGTGCTGCAT** **GGCTGTCGTC**

**Pphosp001**  **TCCAGAGAAT** **TCGCTAGAGA** **TAGCTTAGTG** **CCTTCGGGAA** **CTCTGAGACA** **GGTGCTGCAT** **GGCTGTCGTC**

500 510 520 530 540 550 560

....|....| ....|....| ....|....| ....|....| ....|....| ....|....| ....|....|

**AsalATCC43** **AGCTCGTGTT** **GTGAAATGTT** **GGGTTAAGTC** **CCGCAACGAG** **CGCAACCCTT** **ATCCTTGTTT** **GCCAGCACGT**

**AsalLFl123** **AGCTCGTGTT** **GTGAAATGTT** **GGGTTAAGTC** **CCGCAACGAG** **CGCAACCCTT** **ATCCTTGTTT** **GCCAGCACGT**

**AspR863**  **AGCTCGTGTT** **GTGAAATGTT** **GGGTTAAGTC** **CCGCAACGAG** **CGCAACCCTT** **ATCCTTGTTT** **GCCAGCACGT**

**AspR867**  **AGCTCGTGTT** **GTGAAATGTT** **GGGTTAAGTC** **CCGCAACGAG** **CGCAACCCTT** **ATCCTTGTTT** **GCCAGCACGT**

**VspB925**  **AGCTCGTGTT** **GTGAAATGTT** **GGGTTAAGTC** **CCGCAACGAG** **CGCAACCCTT** **ATCCTTAATT** **GCCAGCGAGT**

**AspB915**  **AGCTCGTGTT** **GTGAAATGTT** **GGGTTAAGTC** **CCGCAACGAG** **CGCAACCCTT** **ATCCTTGTTT** **GCCAGCACGT**

**AwSA12**  **AGCTCGTGTT** **GTGAAATGTT** **GGGTTAAGTC** **CCGCAACGAG** **CGCAACCCTT** **ATCCTTGTTT** **GCCAGCACGT**

**AspMR1766**  **AGCTCGTGTT** **GTGAAATGTT** **GGGTTAAGTC** **CCGCAACGAG** **CGCAACCCTT** **ATCCTTGTTT** **GCCAGCACGT**

**AwATCC1538** **AGCTCGTGTT** **GTGAAATGTT** **GGGTTAAGTC** **CCGCAACGAG** **CGCAACCCTT** **ATCCTTGTTT** **GCCAGCACGT**

**Aw02569**  **AGCTCGTGTT** **GTGAAATGTT** **GGGTTAAGTC** **CCGCAACGAG** **CGCAACCCTT** **ATCCTTGTTT** **GCCAGCACGT**

**Aw01401**  **AGCTCGTGTT** **GTGAAATGTT** **GGGTTAAGTC** **CCGCAACGAG** **CGCAACCCTT** **ATCCTTGTTT** **GCCAGCACGT**

**Aw06139A**  **AGCTCGTGTT** **GTGAAATGTT** **GGGTTAAGTC** **CCGCAACGAG** **CGCAACCCTT** **ATCCTTGTTT** **GCCAGCACGT**

**Al901667**  **AGCTCGTGTT** **GTGAAATGTT** **GGGTTAAGTC** **CCGCAACGAG** **CGCAACCCTT** **ATCCTTGTTT** **GCCAGCACGT**

**Aw03160**  **AGCTCGTGTT** **GTGAAATGTT** **GGGTTAAGTC** **CCGCAACGAG** **CGCAACCCTT** **ATCCTTGTTT** **GCCAGCACGT**

**AwSR6**  **AGCTCGTGTT** **GTGAAATGTT** **GGGTTAAGTC** **CCGCAACGAG** **CGCAACCCTT** **ATCCTTGTTT** **GCCAGCACGT**

**Vt99196**  **AGCTCGTGTT** **GTGAAATGTT** **GGGTTAAGTC** **CCGCAACGAG** **CGCAACCCTT** **ATCCTTGTTT** **GCCAGCGAGT**

**VsplLMG190** **AGCTCGTGTT** **GTGAAATGTT** **GGGTTAAGTC** **CCGCAACGAG** **CGCAACCCTT** **ATCCTTGTTT** **GCCAGCGAGT**

**Vspl02066**  **AGCTCGTGTT** **GTGAAATGTT** **GGGTTAAGTC** **CCGCAACGAG** **CGCAACCCTT** **ATCCTTGTTT** **GCCAGCGAGT**

**Vspl04276**  **AGCTCGTGTT** **GTGAAATGTT** **GGGTTAAGTC** **CCGCAACGAG** **CGCAACCCTT** **ATCCTTGTTT** **GCCAGCGAGT**

**Vspl021491** **AGCTCGTGTT** **GTGAAATGTT** **GGGTTAAGTC** **CCGCAACGAG** **CGCAACCCTT** **ATCCTTGTTT** **GCCAGCGAGT**

**VangNB10**  **AGCTCGTGTT** **GTGAAATGTT** **GGGTTAAGTC** **CCGCAACGAG** **CGCAACCCTT** **ATCCTTGTTT** **GCCAGCGAGT**

**GholLMG177** **AGCTCGTGTT** **GTGAAATGTT** **GGGTTAAGTC** **CCGCAACGAG** **CGCAACCCTT** **ATCCTTGTTT** **GCCAGCACTT**

**VchoATCC14** **AGCTCGTGTT** **GTGAAATGTT** **GGGTTAAGTC** **CCGCAACGAG** **CGCAACCCTT** **ATCCTTGTTT** **GCCAGCACGT**

**VchoO1clas** **AGCTCGTGTT** **GTGAAATGTT** **GGGTTAAGTC** **CCGCAACGAG** **CGCAACCCTT** **ATCCTTGTTT** **GCCAGCACGT**

**VchoO1E1To** **AGCTCGTGTT** **GTGAAATGTT** **GGGTTAAGTC** **CCGCAACGAG** **CGCAACCCTT** **ATCCTTGTTT** **GCCAGCACGT**

**PlumTT01**  **AGCTCGTGTT** **GTGAAATGTT** **GGGTTAAGTC** **CCGCAACGAG** **CGCAACCCTT** **ATCCTTTGTT** **GCCAGCACGT**

**PleiATCC25** **AGCTCGTGTT** **GTGAAATGTT** **GGGTTAAGTC** **CCGCAACGAG** **CGCAACCCTT** **ATCCTTGTTT** **GCCAGCACTT**

**PangATCC25** **AGCTCGTGTT** **GTGAAATGTT** **GGGTTAAGTC** **CCGCAACGAG** **CGCAACCCTT** **ATCCTTGTTT** **GCCAGCACTT**

**PphoATCC11** **AGCTCGTGTT** **GTGAAATGTT** **GGGTTAAGTC** **CCGCAACGAG** **CGCAACCCTT** **ATCCTTGTTT** **GCCAGCACGT**

**AfisES114**  **AGCTCGTGTT** **GTGAAATGTT** **GGGTTAAGTC** **CCGCAACGAG** **CGCAACCCTT** **ATCCTTGTTT** **GCCAGCACGT**

**AfisATCC77** **AGCTCGTGTT** **GTGAAATGTT** **GGGTTAAGTC** **CCGCAACGAG** **CGCAACCCTT** **ATCCTTGTTT** **GCCAGCACGT**

**AfisMJ11**  **AGCTCGTGTT** **GTGAAATGTT** **GGGTTAAGTC** **CCGCAACGAG** **CGCAACCCTT** **ATCCTTGTTT** **GCCAGCACGT**

**AfisSR7**  **AGCTCGTGTT** **GTGAAATGTT** **GGGTTAAGTC** **CCGCAACGAG** **CGCAACCCTT** **ATCCTTGTTT** **GCCAGCACGT**

**AfisATCC25** **AGCTCGTGTT** **GTGAAATGTT** **GGGTTAAGTC** **CCGCAACGAG** **CGCAACCCTT** **ATCCTTGTTT** **GCCAGCACGT**

**AlogATCC29** **AGCTCGTGTT** **GTGAAATGTT** **GGGTTAAGTC** **CCGCAACGAG** **CGCAACCCTT** **ATCCTTGTTT** **GCCAGCACGT**

**AlogWHSW9**  **AGCTCGTGTT** **GTGAAATGTT** **GGGTTAAGTC** **CCGCAACGAG** **CGCAACCCTT** **ATCCTTGTTT** **GCCAGCACGT**

**AlogWHSW1**  **AGCTCGTGTT** **GTGAAATGTT** **GGGTTAAGTC** **CCGCAACGAG** **CGCAACCCTT** **ATCCTTGTTT** **GCCAGCACGT**

**Asif2_4**  **AGCTCGTGTT** **GTGAAATGTT** **GGGTTAAGTC** **CCGCAACGAG** **CGCAACCCTT** **ATCCTTGTTT** **GCCAGCACGT**

**Asif2_5**  **AGCTCGTGTT** **GTGAAATGTT** **GGGTTAAGTC** **CCGCAACGAG** **CGCAACCCTT** **ATCCTTGTTT** **GCCAGCACGT**

**Asif2_6**  **AGCTCGTGTT** **GTGAAATGTT** **GGGTTAAGTC** **CCGCAACGAG** **CGCAACCCTT** **ATCCTTGTTT** **GCCAGCACGT**

**AthoMdR7**  **AGCTCGTGTT** **GTGAAATGTT** **GGGTTAAGTC** **CCGCAACGAG** **CGCAACCCTT** **ATCCTTGTTT** **GCCAGCACGT**

**AthoSA5**  **AGCTCGTGTT** **GTGAAATGTT** **GGGTTAAGTC** **CCGCAACGAG** **CGCAACCCTT** **ATCCTTGTTT** **GCCAGCACGT**

**AthoSA6**  **AGCTCGTGTT** **GTGAAATGTT** **GGGTTAAGTC** **CCGCAACGAG** **CGCAACCCTT** **ATCCTTGTTT** **GCCAGCACGT**

**Pspsp044**  **AGCTCGTGTT** **GTGAAATGTT** **GGGTTAAGTC** **CCGCAACGAG** **CGCAACCCTT** **ATCCTTGTTT** **GCCAGCAC?T**

**Pphosp001**  **AGCTCGTGTT** **GTGAAATGTT** **GGGTTAAGTC** **CCGCAACGAG** **CGCAACCCTT** **ATCCTTGTTT** **GCCAGCACGT**

570 580 590 600 610 620 630

....|....| ....|....| ....|....| ....|....| ....|....| ....|....| ....|....|

**AsalATCC43** **AATGGTGGGA** **ACTCCAGGGA** **GACTGCCGGT** **GATAAACCGG** **AGGAAGGTGG** **GGACGACGTC** **AAGTCATCAT**

**AsalLFl123** **AATGGTGGGA** **ACTCCAGGGA** **GACTGCCGGT** **GATAAACCGG** **AGGAAGGTGG** **GGACGACGTC** **AAGTCATCAT**

**AspR863**  **AATGGTGGGA** **ACTCCAGGGA** **GACTGCCGGT** **GATAAACCGG** **ANGAAGGTGG** **GGACGACGTC** **ANGTCATCAT**

**AspR867**  **AATGGTGGGA** **ACTCCAGGGA** **GACTGCCGGT** **GATAAACCGG** **AGGAAGGTGG** **GGACGACGTC** **AAGTCATCAT**

**VspB925**  **TATGTCGGGA** **ACTTTGGGGA** **GACTGCCGGT** **GATAAACCGG** **AGGAAGGTGG** **GGACGACGTC** **AAGTCATCAT**

**AspB915**  **AATGGTGGGA** **ACTCCAGGGA** **GACTGCCGGT** **GATAAACCGG** **AGGAAGGTGG** **GGACGACGTC** **AAGTCATCAT**

**AwSA12**  **AATGGTGGGA** **ACTCCAGGGA** **GACTGCCGGT** **GATAAACCGG** **AGGAAGGTGG** **GGACGACGTC** **AAGTCATCAT**

**AspMR1766**  **AATGGTGGGA** **ACTCCAGGGA** **GACTGCCGGT** **GATAAACCGG** **AGGAAGGTGG** **GGACGACGTC** **AAGTCATCAT**

**AwATCC1538** **AATGGTGGGA** **ACTCCAGGGA** **GACTGCCGGT** **GATAAACCGG** **AGGAAGGTGG** **GGACGACGTC** **AAGTCATCAT**

**Aw02569**  **AATGGTGGGA** **ACTCCAGGGA** **GACTGCCGGT** **GATAAACCGG** **AGGAAGGTGG** **GGACGACGTC** **AAGTCATCAT**

**Aw01401**  **AATGGTGGGA** **ACTCCAGGGA** **GACTGCCGGT** **GATAAACCGG** **AGGAAGGTGG** **GGACGACGTC** **AAGTCATCAT**

**Aw06139A**  **AATGGTGGGA** **ACTCCAGGGA** **GACTGCCGGT** **GATAAACCGG** **AGGAAGGTGG** **GGACGACGTC** **AAGTCATCAT**

**Al901667**  **AATGGTGGGA** **ACTCCAGGGA** **GACTGCCGGT** **GATAAACCGG** **AGGAAGGTGG** **GGACGACGTC** **AAGTCATCAT**

**Aw03160**  **AATGGTGGGA** **ACTCCAGGGA** **GACTGCCGGT** **GATAAACCGG** **AGGAAGGTGG** **GGACGACGTC** **AAGTCATCAT**

**AwSR6**  **AATGGTGGGA** **ACTCCAGGGA** **GACTGCCGGT** **GATAAACCGG** **AGGAAGGTGG** **GGACGACGTC** **AAGTCATCAT**

**Vt99196**  **AATGTCGGGA** **ACTCCAGGGA** **GACTGCCGGT** **GATAAACCGG** **AGGAAGGTGG** **GGACGACGTC** **AAGTCATCAT**

**VsplLMG190** **AATGTCGGGA** **ACTCCAGGGA** **GACTGCCGGT** **GATAAACCGG** **AGGAAGGTGG** **GGACGACGTC** **AAGTCATCAT**

**Vspl02066**  **AATGTCGGGA** **ACTCCAGGGA** **GACTGCCGGT** **GATAAACCGG** **AGGAAGGTGG** **GGACGACGTC** **AAGTCATCAT**

**Vspl04276**  **AATGTCGGGA** **ACTCCAGGGA** **GACTGCCGGT** **GATAAACCGG** **AGGAAGGTGG** **GGACGACGTC** **AAGTCATCAT**

**Vspl021491** **AATGTCGGGA** **ACTCCAGGGA** **GACTGCCGGT** **GATAAACCGG** **AGGAAGGTGG** **GGACGACGTC** **AAGTCATCAT**

**VangNB10**  **CATGTCGGGA** **ACTCCAGGGA** **GACTGCCGGT** **GATAAACCGG** **AGGAAGGTGG** **GGACGACGTC** **AAGTCATCAT**

**GholLMG177** **CG-GGTGGGA** **ACTCCAGGGA** **GACTGCCGGT** **GATAAACCGG** **AGGAAGGTGG** **GGACGACGTC** **AAGTCATCAT**

**VchoATCC14** **AATGGTGGGA** **ACTCCAGGGA** **GACTGCCGGT** **GATAAACCGG** **AGGAAGGTGG** **GGACGACGTC** **AAGTCATCAT**

**VchoO1clas** **AATGGTGGGA** **ACTCCAGGGA** **GACTGCCGGT** **GATAAACCGG** **AGGAAGGTGG** **GGACGACGTC** **AAGTCATCAT**

**VchoO1E1To** **AATGGTGGGA** **ACTCCAGGGA** **GACTGCCGGT** **GATAAACCGG** **AGGAAGGTGG** **GGACGACGTC** **AAGTCATCAT**

**PlumTT01**  **GAAGGTGGGA** **ACTCAAAGGA** **GACTGCCGGT** **GATAAACCGG** **AGGAAGGTGG** **GGATGACGTC** **AAGTCATCAT**

**PleiATCC25** **CG-GGTGGGA** **ACTCCAGGGA** **GACTGCCGGT** **GATAAACCGG** **AGGAAGGTGG** **GGACGACGTC** **AAGTCATCAT**

**PangATCC25** **ANTGGTGGGA** **ACTCCAGGGA** **GACTGCCGGT** **GATAAACCGG** **AGGAAGGTGG** **GGACGACGTC** **AAGTCATCAT**

**PphoATCC11** **AATGGTGGGA** **ACTCCAGGGA** **GACTGCCGGT** **GATAAACCGG** **AGGAAGGTGG** **GGACGACGTC** **AAGTCATCAT**

**AfisES114**  **AATGGTGGGA** **ACTCCAGGGA** **GACTGCCGGT** **GATAAACCGG** **AGGAAGGTGG** **GGACGACGTC** **AAGTCATCAT**

**AfisATCC77** **AATGGTGGGA** **ACTCCAGGGA** **GACTGCCGGT** **GATAAACCGG** **AGGAAGGTGG** **GGACGACGTC** **AAGTCATCAT**

**AfisMJ11**  **AATGGTGGGA** **ACTCCAGGGA** **GACTGCCGGT** **GATAAACCGG** **AGGAAGGTGG** **GGACGACGTC** **AAGTCATCAT**

**AfisSR7**  **AATGGTGGGA** **ACTCCAGGGA** **GACTGCCGGT** **GATAAACCGG** **AGGAAGGTGG** **GGACGACGTC** **AAGTCATCAT**

**AfisATCC25** **AATGGTGGGA** **ACTCCAGGGA** **GACTGCCGGT** **GATAAACCGG** **AGGAAGGTGG** **GGACGACGTC** **AAGTCATCAT**

**AlogATCC29** **AATGGTGGGA** **ACTCCAGGGA** **GACTGCCGGT** **GATAAACCGG** **AGGAAGGTGG** **GGACGACGTC** **AAGTCATCAT**

**AlogWHSW9**  **AATGGTGGGA** **ACTCCAGGGA** **GACTGCCGGT** **GATAAACCGG** **AGGAAGGTGG** **GGACGACGTC** **AAGTCATCAT**

**AlogWHSW1**  **AATGGTGGGA** **ACTCCAGGGA** **GACTGCCGGT** **GATAAACCGG** **AGGAAGGTGG** **GGACGACGTC** **AAGTCATCAT**

**Asif2_4**  **AATGGTGGGA** **ACTCCAGGGA** **GACTGCCGGT** **GATAAACCGG** **AGGAAGGTGG** **GGACGACGTC** **AAGTCATCAT**

**Asif2_5**  **AATGGTGGGA** **ACTCCAGGGA** **GACTGCCGGT** **GATAAACCGG** **AGGAAGGTGG** **GGACGACGTC** **AAGTCATCAT**

**Asif2_6**  **AATGGTGGGA** **ACTCCAGGGA** **GACTGCCGGT** **GATAAACCGG** **AGGAAGGTGG** **GGACGACGTC** **AAGTCATCAT**

**AthoMdR7**  **AATGGTGGGA** **ACTCCAGGGA** **GACTGCCGGT** **GATAAACCGG** **AGGAAGGTGG** **GGACGACGTC** **AAGTCATCAT**

**AthoSA5**  **AATGGTGGGA** **ACTCCAGGGA** **GACTGCCGGT** **GATAAACCGG** **AGGAAGGTGG** **GGACGACGTC** **AAGTCATCAT**

**AthoSA6**  **AATGGTGGGA** **ACTCCAGGGA** **GACTGCCGGT** **GATAAACCGG** **AGGAAGGTGG** **GGACGACGTC** **AAGTCATCAT**

**Pspsp044**  **AATGGTGGGA** **ACTCCAGGGA** **GACTGCCGGT** **GATAAACCGG** **AGGAAGGTGG** **GGACGACGTC** **AAGTCATCAT**

**Pphosp001**  **AATGGTGGGA** **ACTCCAGGGA** **GACTGCCGGT** **GATAAACCGG** **AGGAAGGTGG** **GGACGACGTC** **AAGTCATCAT**

640 650 660 670 680 690 700

....|....| ....|....| ....|....| ....|....| ....|....| ....|....| ....|....|

**AsalATCC43** **GGCCCTTACG** **AGTAGGGCTA** **CACACGTGCT** **ACAATGGCGC** **ATACAGAGGG** **CTGCAAGCTA** **GCGATAGTGA**

**AsalLFl123** **GGCCCTTACG** **AGTAGGGCTA** **CACACGTGCT** **ACAATGGCGC** **ATACAGAGGG** **CTGCAAGCTA** **GCGATAGTGA**

**AspR863**  **GGCCCTTACG** **AGTAGGGCTA** **CACACGTGCT** **ACA??????? ?????????? ?????????? ??????????**

**AspR867**  **GGCCCTTACG** **AGTAGGGCTA** **CACACGTGCT** **ACAATGGCGT** **ATACAGAGGG** **CTGCAAGCTA** **GCGATAGTGA**

**VspB925**  **GGCCCTTACG** **AGTAGGGCTA** **CACACGTGCT** **ACAATGGCGC** **ATACAGAGGG** **CAGCCAGCTA** **GCAATAGTGA**

**AspB915**  **GGCCCTTACG** **AGTAGGGCTA** **CACACGTGCT** **ACAATGGCGC** **ATACAGAGGG** **CTGCAAGCTA** **GCGATAGTGA**

**AwSA12**  **GGCCCTTACG** **AGTAGGGCTA** **CACACGTGCT** **ACAATGGCGC** **ATACAGAGGG** **CTGCAAGCTA** **GCGATAGTGA**

**AspMR1766**  **GGCCCTTACG** **AGTAGGGCTA** **CACACGTGCT** **ACAATGGCGC** **ATACAGAGGG** **CTGCAAGCTA** **GCGATAGTGA**

**AwATCC1538** **GGCCCTTACG** **AGTAGGGCTA** **CACACGTGCT** **ACAATGGCGC** **ATACAGAGGG** **CTGCAAGCTA** **GCGATAGTGA**

**Aw02569**  **GGCCCTTACG** **AGTAGGGCTA** **CACACGTGCT** **ACAATGGCGC** **ATACAGAGGG** **CTGCAAGCTA** **GCGATAGTGA**

**Aw01401**  **GGCCCTTACG** **AGTAGGGCTA** **CACACGTGCT** **ACAATGGCGC** **ATACAGAGGG** **CTGCAAGCTA** **GCGATAGTGA**

**Aw06139A**  **GGCCCTTACG** **AGTAGGGCTA** **CACACGTGCT** **ACAATGGCGC** **ATACAGAGGG** **CTGCAAGCTA** **GCGATAGTGA**

**Al901667**  **GGCCCTTACG** **AGTAGGGCTA** **CACACGTGCT** **ACAATGGCGC** **ATACAGAGGG** **CTGCAAGCTA** **GCGATAGTGA**

**Aw03160**  **GGCCCTTACG** **AGTAGGGCTA** **CACACGTGCT** **ACAATGGCGC** **ATACAGAGGG** **CTGCAAGCTA** **GCGATAGTGA**

**AwSR6**  **GGCCCTTACG** **AGTAGGGCTA** **CACACGTGCT** **ACAATGGCGC** **ATACAGAGGG** **CTGCAAGCTA** **GCGATAGTGA**

**Vt99196**  **GGCCCTTACG** **AGTAGGGCTA** **CACACGTGCT** **ACAATGGCGC** **ATACAGAGGG** **CAGCCAACCA** **GCGATGGTGA**

**VsplLMG190** **GGCCCTTACG** **AGTAGGGCTA** **CACACGTGCT** **ACAATGGCGC** **ATACAGAGGG** **CAGCAAGCTA** **GCGATAGTGA**

**Vspl02066**  **GGCCCTTACG** **AGTAGGGCTA** **CACACGTGCT** **ACAATGGCGC** **ATACAGAGGG** **CAGCAAGCTA** **GCGATAGTGA**

**Vspl04276**  **GGCCCTTACG** **AGTAGGGCTA** **CACACGTGCT** **ACAATGGCGC** **ATACAGAGGG** **CAGCAAGCTA** **GCGATAGTGA**

**Vspl021491** **GGCCCTTACG** **AGTAGGGCTA** **CACACGTGCT** **ACAATGGCGC** **ATACAGAGGG** **CAGCAAGCTA** **GCGATAGTGA**

**VangNB10**  **GGCCCTTACG** **AGTAGGGCTA** **CACACGTGCT** **ACAATGGCGC** **ATACAGAGGG** **CAGCAAGCTA** **GCGATAGTGA**

**GholLMG177** **GGCCCTTACG** **AGTAGGGCTA** **CACACGTGCT** **ACAATGGTGC** **ATACAGAGGG** **CAGCGAGACC** **GCGAGGTGGA**

**VchoATCC14** **GGCCCTTACG** **AGTAGGGCTA** **CACACGTGCT** **ACAATGGCGT** **ATACAGAGGG** **CAGCGATACC** **GCGAGGTGGA**

**VchoO1clas** **GGCCCTTACG** **AGTAGGGCTA** **CACACGTGCT** **ACAATGGCGT** **ATACAGAGGG** **CAGCGATACC** **GCGAGGTGGA**

**VchoO1E1To** **GGCCCTTACG** **AGTAGGGCTA** **CACACGTGCT** **ACAATGGCGT** **ATACAGAGGG** **CAGCGATACC** **GCGAGGTGGA**

**PlumTT01**  **GGCCCTTACG** **AGTAGGGCTA** **CACACGTGCT** **ACAATGGCGG** **ATACAAAGTG** **AAGCGACCTC** **GCGAGAGCAA**

**PleiATCC25** **GGCCCTTACG** **AGTAGGGCTA** **CACACGTGCT** **ACAATGGCGT** **ATACAGAGGG** **CTGCCAACTC** **GCGAGAGTGA**

**PangATCC25** **GGCCCTTACG** **AGTAGGGCTA** **CACACGTGCT** **ACAATGGCGT** **ATACAGAGGG** **CTGCCAACTA** **GCGATAGTGA**

**PphoATCC11** **GGCCCTTACG** **AGTAGGGCTA** **CACACGTGCT** **ACAATGGCGT** **ATACAGAGGG** **CTGCAAGCTA** **GCGATAGT?A**

**AfisES114**  **GGCCCTTACG** **AGTAGGGCTA** **CACACGTGCT** **ACAATGGCGC** **ATACAGAGGG** **CTGCAAGCTA** **GCGATAGTGA**

**AfisATCC77** **GGCCCTTACG** **AGTAGGGCTA** **CACACGTGCT** **ACAATGGCGC** **ATACAGAGGG** **CTGCAAGCTA** **GCGATAGTGA**

**AfisMJ11**  **GGCCCTTACG** **AGTAGGGCTA** **CACACGTGCT** **ACAATGGCGC** **ATACAGAGGG** **CTGCAAGCTA** **GCGATAGTGA**

**AfisSR7**  **GGCCCTTACG** **AGTAGGGCTA** **CACACGTGCT** **ACAATGGCGC** **ATACAGAGGG** **CTGCAAGCTA** **GCGATAGTGA**

**AfisATCC25** **GGCCCTTACG** **AGTAGGGCTA** **CACACGTGCT** **ACAATGGCGC** **ATACAGAGGG** **CTGCAAGCTA** **GCGATAGTGA**

**AlogATCC29** **GGCCCTTACG** **AGTAGGGCTA** **CACACGTGCT** **ACAATGGCGC** **ATACAGAGGG** **CTGCAAGCTA** **GCGATAGTGA**

**AlogWHSW9**  **GGCCCTTACG** **AGTAGGGCTA** **CACACGTGCT** **ACAATGGCGC** **ATACAGAGGG** **CTGCAAGCTA** **GCGATAGTGA**

**AlogWHSW1**  **GGCCCTTACG** **AGTAGGGCTA** **CACACGTGCT** **ACAATGGCGC** **ATACAGAGGG** **CTGCAAGCTA** **GCGATAGTGA**

**Asif2_4**  **GGCCCTTACG** **AGTAGGGCTA** **CACACGTGCT** **ACAATGGCGC** **ATACAGAGGG** **CTGCAAGCTA** **GCGATAGTGA**

**Asif2_5**  **GGCCCTTACG** **AGTAGGGCTA** **CACACGTGCT** **ACAATGGCGC** **ATACAGAGGG** **CTGCAAGCTA** **GCGATAGTGA**

**Asif2_6**  **GGCCCTTACG** **AGTAGGGCTA** **CACACGTGCT** **ACAATGGCGC** **ATACAGAGGG** **CTGCAAGCTA** **GCGATAGTGA**

**AthoMdR7**  **GGCCCTTACG** **AGTAGGGCTA** **CACACGTGCT** **ACAATGGCGC** **ATACAGAGGG** **C?GCAA?CTA** **GCGATAGT?A**

**AthoSA5**  **GGCCCTTACG** **AGTAGGGCTA** **CACACGTGCT** **ACAATGGCGC** **ATACAGAGGG** **C?GCAA?CTA** **GCGATAGT?A**

**AthoSA6**  **GGCCCTTACG** **AGTAGGGCTA** **CACACGTGCT** **ACAATGGCGC** **ATACAGAGGG** **C?GCAA?CTA** **GCGATAGT?A**

**Pspsp044**  **GGCCCTTACG** **AGTAGGGCTA** **CACACGTGCT** **ACAATGGCGT** **ATACAGAGGG** **CTGCCAACCA** **GCGATGGTGA**

**Pphosp001**  **GGCCCTTACG** **AGTAGGGCTA** **CACACGTGCT** **ACAATGGCGT** **ATACAGAGGG** **CTGCAAGCTA** **GCGATAGTGA**

710 720 730 740 750 760 770

....|....| ....|....| ....|....| ....|....| ....|....| ....|....| ....|....|

**AsalATCC43** **GCGAATCCCA** **AAAAGTGCGT** **CGTAGTCCGG** **ATCGGAGTCT** **GCAACTCGAC** **TCCGTGAAGT** **CGGAATCGCT**

**AsalLFl123** **GCGAATCCCA** **AAAAGTGCGT** **CGTAGTCCGG** **ATCGGAGTCT** **GCAACTCGAC** **TCCGTGAAGT** **CGGAATCGCT**

**AspR863**  ?????????? ?????????? ?????????? ?????????? ?????????? ?????????? ??????????

**AspR867**  **GCGAATCCCA** **AAAAGTACGT** **CGTAGTCCGG** **ATCGGAGTCT** **GCAACTCGAC** **TCCGTGAAGT** **CGGAATCGCT**

**VspB925**  **GCGAATCCCA** **AAAAGTGCGT** **CGTAGTCCGG** **ATTGGAGTCT** **GCAACTCGAC** **TCCATGAAGT** **CGGAATCGCT**

**AspB915**  **GCGAATCCCA** **AAAAGTGCGT** **CGTAGTCCGG** **ATCGGAGTCT** **GCAACTCGAC** **TCCGTGAAGT** **CGGAATCGCT**

**AwSA12**  **GCGAATCCCA** **AAAAGTGCGT** **CGTAGTCCGG** **ATCGGAGTCT** **GCAACTCGAC** **TCCGTGAAGT** **CGGAATCGCT**

**AspMR1766**  **GCGAATCCCA** **AAAAGTGCGT** **CGTAGTCCGG** **ATCGGAGTCT** **GCAACTCGAC** **TCCGTGAAGT** **CGGAATCGCT**

**AwATCC1538** **GCGAATCCCA** **AAAAGTGCGT** **CGTAGTCCGG** **ATCGGAGTCT** **GCAACTCGAC** **TCCGTGAAGT** **CGGAATCGCT**

**Aw02569**  **GCGAATCCCA** **AAAAGTGCGT** **CGTAGTCCGG** **ATCGGAGTCT** **GCAACTCGAC** **TCCGTGAAGT** **CGGAATCGCT**

**Aw01401**  **GCGAATCCCA** **AAAAGTGCGT** **CGTAGTCCGG** **ATCGGAGTCT** **GCAACTCGAC** **TCCGTGAAGT** **CGGAATCGCT**

**Aw06139A**  **GCGAATCCCA** **AAAAGTGCGT** **CGTAGTCCGG** **ATCGGAGTCT** **GCAACTCGAC** **TCCGTGAAGT** **CGGAATCGCT**

**Al901667**  **GCGAATCCCA** **AAAAGTGCGT** **CGTAGTCCGG** **ATCGGAGTCT** **GCAACTCGAC** **TCCGTGAAGT** **CGGAATCGCT**

**Aw03160**  **GCGAATCCCA** **AAAAGTGCGT** **CGTAGTCCGG** **ATCGGAGTCT** **GCAACTCGAC** **TCCGTGAAGT** **CGGAATCGCT**

**AwSR6**  **GCGAATCCCA** **AAAAGTGCGT** **CGTAGTCCGG** **ATCGGAGTCT** **GCAACTCGAC** **TCCGTGAAGT** **CGGAATCGCT**

**Vt99196**  **GCGAATCCCA** **AAAAGTGCGT** **CGTAGTCCGG** **ATTGGAGTCT** **GCAACTCGAC** **TCCATGAAGT** **CGGAATCGCT**

**VsplLMG190** **GCGAATCCCA** **AAAAGTGCGT** **CGTAGTCCGG** **ATTGGAGTCT** **GCAACTCGAC** **TCCATGAAGT** **CGGAATCGCT**

**Vspl02066**  **GCGAATCCCA** **AAAAGTGCGT** **CGTAGTCCGG** **ATTGGAGTCT** **GCAACTCGAC** **TCCATGAAGT** **CGGAATCGCT**

**Vspl04276**  **GCGAATCCCA** **AAAAGTGCGT** **CGTAGTCCGG** **ATTGGAGTCT** **GCAACTCGAC** **TCCATGAAGT** **CGGAATCGCT**

**Vspl021491** **GCGAATCCCA** **AAAAGTGCGT** **CGTAGTCCGG** **ATTGGAGTCT** **GCAACTCGAC** **TCCATGAAGT** **CGGAATCGCT**

**VangNB10**  **GCGAATCCCA** **AAAAGTGCGT** **CGTAGTCCGG** **ATTGGAGTCT** **GCAACTCGAC** **TCCATGAAGT** **CGGAATCGCT**

**GholLMG177** **GCGAATCCCA** **GAAAGTGCAT** **CGTAGTCCGG** **ATTGGAGTCT** **GCAACTCGAC** **TCCATGAAGT** **CGGAATCGCT**

**VchoATCC14** **GCGAATCTCA** **CAAAGTACGT** **CGTAGTCCGG** **ATTGGAGTCT** **GCAACTCGAC** **TCCATGAAGT** **CGGAATCGCT**

**VchoO1clas** **GCGAATCTCA** **CAAAGTACGT** **CGTAGTCCGG** **ATTGGAGTCT** **GCAACTCGAC** **TCCATGAAGT** **CGGAATCGCT**

**VchoO1E1To** **GCGAATCTCA** **CAAAGTACGT** **CGTAGTCCGG** **ATTGGAGTCT** **GCAACTCGAC** **TCCATGAAGT** **CGGAATCGCT**

**PlumTT01**  **GCGGAACACA** **CAAAGTCTGT** **CGTAGTCCGG** **ATTGGAGTCT** **GCAACTCGAC** **TCCATGAAGT** **CGGAATCGCT**

**PleiATCC25** **GCGAATCCCA** **GAAAGTACGT** **CGTAGTCCGG** **ATTGGAGTCT** **GCAACTCGAC** **TCCATGAAGT** **CGGAATCGCT**

**PangATCC25** **GCGAATCCCA** **GAAAGTACGT** **CGTAGTCCGG** **ATTGGAGTCT** **GCAACTCGAC** **TCCATGAAGT** **CGGAATCGCT**

**PphoATCC11** **GCGAATCCCA** **CAAAGTACGT** **CGTAGTCCGG** **ATTGGAGTCT** **GCAACTCGAC** **TCCATGAAGT** **CGGAATCGCT**

**AfisES114**  **GCGAATCCCA** **AAAAGTGCGT** **CGTAGTCCGG** **ATTGGAGTCT** **GCAACTCGAC** **TCCATGAAGT** **CGGAATCGCT**

**AfisATCC77** **GCGAATCCCA** **AAAAGTGCGT** **CGTAGTCCGG** **ATTGGAGTCT** **GCAACTCGAC** **TCCATGAAGT** **CGGAATCGCT**

**AfisMJ11**  **GCGAATCCCA** **AAAAGTGCGT** **CGTAGTCCGG** **ATTGGAGTCT** **GCAACTCGAC** **TCCATGAAGT** **CGGAATCGCT**

**AfisSR7**  **GCGAATCCCA** **AAAAGTGCGT** **CGTAGTCCGG** **ATTGGAGTCT** **GCAACTCGAC** **TCCATGAAGT** **CGGAATCGCT**

**AfisATCC25** **GCGAATCCCA** **AAAAGTGCGT** **CGTAGTCCGG** **ATTGGAGTCT** **GCAACTCGAC** **TCCATGAAGT** **CGGAATCGCT**

**AlogATCC29** **GCGAATCCCA** **AAAAGTGCGT** **CGTAGTCCGG** **ATCGGAGTCT** **GCAACTCGAC** **TCCGTGAAGT** **CGGAATCGCT**

**AlogWHSW9**  **GCGAATCCCA** **AAAAGTGCGT** **CGTAGTCCGG** **ATCGGAGTCT** **GCAACTCGAC** **TCCGTGAAGT** **CGGAATCGCT**

**AlogWHSW1**  **GCGAATCCCA** **AAAAGTGCGT** **CGTAGTCCGG** **ATCGGAGTCT** **GCAACTCGAC** **TCCGTGAAGT** **CGGAATCGCT**

**Asif2_4**  **GCGAATCCCA** **AAAAGTGCGT** **CGTAGTCCGG** **ATCGGAGTCT** **GCAACTCGAC** **TCCGTGAAGT** **CGGAATCGCT**

**Asif2_5**  **GCGAATCCCA** **AAAAGTGCGT** **CGTAGTCCGG** **ATCGGAGTCT** **GCAACTCGAC** **TCCGTGAAGT** **CGGAATCGCT**

**Asif2_6**  **GCGAATCCCA** **AAAAGTGCGT** **CGTAGTCCGG** **ATCGGAGTCT** **GCAACTCGAC** **TCCGTGAAGT** **CGGAATCGCT**

**AthoMdR7**  **GCGAATCCCA** **AAAAGTGCGT** **CGTAGTCCGG** **ATCGGAGTCT** **GCAACTCGAC** **TCCGT?AAGT** **CGGAATCGCT**

**AthoSA5**  **GCGAATCCCA** **AAAAGTGCGT** **CGTAGTCCGG** **ATCGGAGTCT** **GCAACTCGAC** **TCCGTGAAGT** **CGGAATCGCT**

**AthoSA6**  **GCGAATCCCA** **AAAAGTGCGT** **CGTAGTCCGG** **ATCGGAGTCT** **GCAACTCGAC** **TCCGTGAAGT** **CGGAATCGCT**

**Pspsp044**  **GCGAATCCCA** **CAAAGTACGT** **CGTAGTCCGG** **ATCGGAGTCT** **GCAACTCGAC** **TCCGTGAAGT** **CGGAATCGCT**

**Pphosp001**  **GCGAATCCCA** **CAAAGTACGT** **CGTAGTCCGG** **ATTGGAGTCT** **GCAACTCGAC** **TCCATGAAGT** **CGGAATCGCT**

780 790 800 810 820 830 840

....|....| ....|....| ....|....| ....|....| ....|....| ....|....| ....|....|

**AsalATCC43** **AGTAATCGTG** **AATCAGAATG** **TCACGGTGAA** **TACGTTCCCG** **GGCCTTGTAC** **ACACCGCCCG** **TCACACCATG**

**AsalLFl123** **AGTAATCGTG** **AATCAGAATG** **TCACGGTGAA** **TACGTTCCCG** **GGCCTTGTAC** **ACACCGCCCG** **TCACACCATG**

**AspR863**  ?????????? ?????????? ?????????? ?????????? ?????????? ?????????? ??????????

**AspR867**  **AGTAATCGTG** **AATCAGAATG** **TCACGGTGAA** **TACGTTCCCG** **GGCCTTGTAC** **ACACCGCCCG** **TCACACCATG**

**VspB925**  **AGTAATCGTA** **GATCAGAATG** **CTACGGTGAA** **TACGTTCCCG** **GGCCTTGTAC** **ACACCGCCCG** **TCACACCATG**

**AspB915**  **AGTAATCGTG** **AATCAGAATG** **TCACGGTGAA** **TACGTTCCCG** **GGCCTTGTAC** **ACACCGCCCG** **TCACACCATG**

**AwSA12**  **AGTAATCGTG** **AATCAGAATG** **TCACGGTGAA** **TACGTTCCCG** **GGCCTTGTAC** **ACACCGCCCG** **TCACACCATG**

**AspMR1766**  **AGTAATCGTG** **AATCAGAATG** **TCACGGTGAA** **TACGTTCCCG** **GGCCTTGTAC** **ACACCGCCCG** **TCACACCATG**

**AwATCC1538** **AGTAATCGTG** **AATCAGAATG** **TCACGGTGAA** **TACGTTCCCG** **GGCCTTGTAC** **ACACCGCCCG** **TCACACCATG**

**Aw02569**  **AGTAATCGTG** **AATCAGAATG** **TCACGGTGAA** **TACGTTCCCG** **GGCCTTGTAC** **ACACCGCCCG** **TCACACCATG**

**Aw01401**  **AGTAATCGTG** **AATCAGAATG** **TCACGGTGAA** **TACGTTCCCG** **GGCCTTGTAC** **ACACCGCCCG** **TCACACCATG**

**Aw06139A**  **AGTAATCGTG** **AATCAGAATG** **TCACGGTGAA** **TACGTTCCCG** **GGCCTTGTAC** **ACACCGCCCG** **TCACACCATG**

**Al901667**  **AGTAATCGTG** **AATCAGAATG** **TCACGGTGAA** **TACGTTCCCG** **GGCCTTGTAC** **ACACCGCCCG** **TCACACCATG**

**Aw03160**  **AGTAATCGTG** **AATCAGAATG** **TCACGGTGAA** **TACGTTCCCG** **GGCCTTGTAC** **ACACCGCCCG** **TCACACCATG**

**AwSR6**  **AGTAATCGTG** **AATCAGAATG** **TCACGGTGAA** **TACGTTCCCG** **GGCCTTGTAC** **ACACCGCCCG** **TCACACCATG**

**Vt99196**  **AGTAATCGTG** **AATCAGAATG** **TCACGGTGAA** **TACGTTCCCG** **GGCCTTGTAC** **ACACCGCCCG** **TCACACCATG**

**VsplLMG190** **AGTAATCGTG** **AATCAGAATG** **TCACGGTGAA** **TACGTTCCCG** **GGCCTTGTAC** **ACACCGCCCG** **TCACACCATG**

**Vspl02066**  **AGTAATCGTG** **AATCAGAATG** **TCACGGTGAA** **TACGTTCCCG** **GGCCTTGTAC** **ACACCGCCCG** **TCACACCATG**

**Vspl04276**  **AGTAATCGTG** **AATCAGAATG** **TCACGGTGAA** **TACGTTCCCG** **GGCCTTGTAC** **ACACCGCCCG** **TCACACCATG**

**Vspl021491** **AGTAATCGTG** **AATCAGAATG** **TCACGGTGAA** **TACGTTCCCG** **GGCCTTGTAC** **ACACCGCCCG** **TCACACCATG**

**VangNB10**  **AGTAATCGTA** **GATCAGAATG** **CTACGGTGAA** **TACGTTCCCG** **GGCCTTGTAC** **ACACCGCCCG** **TCACACCATG**

**GholLMG177** **AGTAATCGTG** **GATCAGAATG** **CCACGGTGAA** **TACGTTCCCG** **GGCCTTGTAC** **ACACCGCCCG** **TCACACCATG**

**VchoATCC14** **AGTAATCGCA** **AATCAGAATG** **TTGCGGTGAA** **TACGTTCCCG** **GGCCTTGTAC** **ACACCGCCCG** **TCACACCATG**

**VchoO1clas** **AGTAATCGCA** **AATCAGAATG** **TTGCGGTGAA** **TACGTTCCCG** **GGCCTTGTAC** **ACACCGCCCG** **TCACACCATG**

**VchoO1E1To** **AGTAATCGCA** **AATCAGAATG** **TTGCGGTGAA** **TACGTTCCCG** **GGCCTTGTAC** **ACACCGCCCG** **TCACACCATG**

**PlumTT01**  **AGTAATCGTA** **GATCAGCATG** **CTACGGTGAA** **TACGTTCCCG** **GGCCTTGTAC** **ACACCGCCCG** **TCACACCATG**

**PleiATCC25** **AGTAATCGTG** **AATCAGAATG** **TCACGGTGAA** **TACGTTCCCG** **GGCCTTGTAC** **ACACCGCCCG** **TCACACCATG**

**PangATCC25** **AGTAATCGTG** **AATCAGAATG** **TCACGGTGAA** **TACGTTCCCG** **GGCCTTGTAC** **ACACCGCCCG** **TCACACCATG**

**PphoATCC11** **AGTAATCGTG** **AATCAGAATG** **TCACGGTGAA** **TACGTTCCCG** **GGCCTTGTAC** **ACACCGCCCG** **TCACACCATG**

**AfisES114**  **AGTAATCGTA** **GATCAGAATG** **CTACGGTGAA** **TACGTTCCCG** **GGCCTTGTAC** **ACACCGCCCG** **TCACACCATG**

**AfisATCC77** **AGTAATCGTA** **GATCAGAATG** **CTACGGTGAA** **TACGTTCCCG** **GGCCTTGTAC** **ACACCGCCCG** **TCACACCATG**

**AfisMJ11**  **AGTAATCGTA** **GATCAGAATG** **CTACGGTGAA** **TACGTTCCCG** **GGCCTTGTAC** **ACACCGCCCG** **TCACACCATG**

**AfisSR7**  **AGTAATCGTA** **GATCAGAATG** **CTACGGTGAA** **TACGTTCCCG** **GGCCTTGTAC** **ACACCGCCCG** **TCACACCATG**

**AfisATCC25** **AGTAATCGTA** **GATCAGAATG** **CTACGGTGAA** **TACGTTCCCG** **GGCCTTGTAC** **ACACCGCCCG** **TCACACCATG**

**AlogATCC29** **AGTAATCGTG** **AATCAGAATG** **TCACGGTGAA** **TACGTTCCCG** **GGCCTTGTAC** **ACACCGCCCG** **TCACACCATG**

**AlogWHSW9**  **AGTAATCGTG** **AATCAGAATG** **TCACGGTGAA** **TACGTTCCCG** **GGCCTTGTAC** **ACACCGCCCG** **TCACACCATG**

**AlogWHSW1**  **AGTAATCGTG** **AATCAGAATG** **TCACGGTGAA** **TACGTTCCCG** **GGCCTTGTAC** **ACACCGCCCG** **TCACACCATG**

**Asif2_4**  **AGTAATCGTG** **AATCAGAATG** **TCACGGTGAA** **TACGTTCCCG** **GGCCTTGTAC** **ACACCGCCCG** **TCACACCATG**

**Asif2_5**  **AGTAATCGTG** **AATCAGAATG** **TCACGGTGAA** **TACGTTCCCG** **GGCCTTGTAC** **ACACCGCCCG** **TCACACCATG**

**Asif2_6**  **AGTAATCGTG** **AATCAGAATG** **TCACGGTGAA** **TACGTTCCCG** **GGCCTTGTAC** **ACACCGCCCG** **TCACACCATG**

**AthoMdR7**  **AGTAATCGT?** **?ATCAGAATG** **??ACGGTGAA** **TACGTTCCCG** **GGCCTTGTAC** **ACACCGCCCG** **TCACACCATG**

**AthoSA5**  **AGTAATCGT?** **?ATCAGAATG** **??ACGGTGAA** **TACGTTCCCG** **GGCCTTGTAC** **ACACCGCCCG** **TCACACCATG**

**AthoSA6**  **AGTAATCGT?** **?ATCAGAATG** **??ACGGTGAA** **TACGTTCCCG** **GGCCTTGTAC** **ACACCGCCCG** **TCACACCATG**

**Pspsp044**  **AGTAATCGTG** **AATCAGAATG** **TCACGGTGAA** **TACGTTCCCG** **GGCCTTGTAC** **ACACCGCCCG** **TCACACCATG**

**Pphosp001**  **AGTAATCGTG** **AATCAGAATG** **TCACGGTGAA** **TACGTTCCCG** **GGCCTTGTAC** **ACACCGCCCG** **TCACACCATG**

850

....|....| ..

**AsalATCC43** **GGAGTGGGCT** **GC**

**AsalLFl123** **GGAGTGGGCT** **GC**

**AspR863**  ?????????? ??

**AspR867**  **GGAGTGGGCT** **GC**

**VspB925**  **GGAGTGGGCT** **GC**

**AspB915**  **GGAGTGGGCT** **GC**

**AwSA12**  **GGAGTGGGCT** **GC**

**AspMR1766**  **GGAGTGGGCT** **GC**

**AwATCC1538** **GGAGTGGGCT** **GC**

**Aw02569**  **GGAGTGGGCT** **GC**

**Aw01401**  **GGAGTGGGCT** **GC**

**Aw06139A**  **GGAGTGGGCT** **GC**

**Al901667**  **GGAGTGGGCT** **GC**

**Aw03160**  **GGAGTGGGCT** **GC**

**AwSR6**  **GGAGTGGGCT** **GC**

**Vt99196**  **GGAGTGGGCT** **GC**

**VsplLMG190** **GGAGTGGGCT** **GC**

**Vspl02066**  **GGAGTGGGCT** **GC**

**Vspl04276**  **GGAGTGGGCT** **GC**

**Vspl021491** **GGAGTGGGCT** **GC**

**VangNB10**  **GGAGTGGGCT** **GC**

**GholLMG177** **GGAGTGGGCT** **GC**

**VchoATCC14** **GGAGTGGGCT** **GC**

**VchoO1clas** **GGAGTGGGCT** **GC**

**VchoO1E1To** **GGAGTGGGCT** **GC**

**PlumTT01**  **GGAGTGGGTT** **GC**

**PleiATCC25** **GGAGTGGGCT** **GC**

**PangATCC25** **GGAGTGGGCT** **GC**

**PphoATCC11** **GGAGTGGGCT** **GC**

**AfisES114**  **GGAGTGGGCT** **GC**

**AfisATCC77** **GGAGTGGGCT** **GC**

**AfisMJ11**  **GGAGTGGGCT** **GC**

**AfisSR7**  **GGAGTGGGCT** **GC**

**AfisATCC25** **GGAGTGGGCT** **GC**

**AlogATCC29** **GGAGTGGGCT** **GC**

**AlogWHSW9**  **GGAGTGGGCT** **GC**

**AlogWHSW1**  **GGAGTGGGCT** **GC**

**Asif2_4**  **GGAGTGGGCT** **GC**

**Asif2_5**  **GGAGTGGGCT** **GC**

**Asif2_6**  **GGAGTGGGCT** **GC**

**AthoMdR7**  **GGAGTGGGCT** **GC**

**AthoSA5**  **GGAGTGGGCT** **GC**

**AthoSA6**  **GGAGTGGGCT** **GC**

**Pspsp044**  **GGAGTGGGCT** **GC**

**Pphosp001**  **GGAGTGGGCT** **GC**
